# The Role of Attachment Trauma and Disintegrative Pathogenic Processes in the Traumatic-Dissociative Dimension

**DOI:** 10.3389/fpsyg.2019.00933

**Published:** 2019-04-26

**Authors:** Benedetto Farina, Marianna Liotti, Claudio Imperatori

**Affiliations:** ^1^ Department of Human Sciences, European University of Rome, Rome, Italy; ^2^ Traumatic Treatment Unit, Centro Clinico De Sanctis, Rome, Italy; ^3^ Italian School of Clinical Cognitivism, Rome, Italy

**Keywords:** developmental trauma, complex PTSD, traumatic attachment, dissociation, emotional dysregulation, traumatic-dissociative dimension

## Abstract

Epidemiological, clinical, and neurobiological studies of the last 30 years suggest that traumatic attachments during the early years of life are associated to specific psychopathological vulnerabilities based on dissociative pathogenic processes. It has been observed that the dissociative pathogenic processes caused by these traumatic attachments either may contribute to the genesis of well-defined mental disorders (e.g., dissociative disorders) or may variably occur in many other diagnostic categories, complicating their clinical pictures and worsening their prognosis. For this reason, we proposed to define the dimension of psychopathological outcomes linked to traumatic attachments and dissociative pathogenic processes as the “traumatic-dissociative” dimension (TDD). The clinical complexity of the TDD requires specific training to enable mental health professionals to recognize the signs of traumatic developments and to implement specific treatment strategies. The present article aims to review some crucial points about the clinical meaning and treatment strategies of the TDD, the dissociative pathogenic processes characterizing the TDD, as well as of the role of attachment trauma in the TDD. We also focused on the clinical and theoretical evidence suggesting that dissociation and dis-integration may be considered two different processes but highly correlated. The usefulness of clinical reasoning in terms of psychopathological dimensions, instead of distinct diagnostic categories, as well as several therapeutic implications of these issues was finally discussed.

## Introduction

Over the last 30 years, an increasing body of experimental data highlighted both the importance of developmental trauma and, at the same time, the difficulty of current international diagnostic systems in describing the clinical consequences of cumulative traumatic childhood experiences in the adult population (Van der Kolk et al., 1996; Cloitre et al., 2009; Bryant, 2010). Cumulative developmental trauma (CDT), also known as early relational trauma due to the interpersonal nature of the traumatic experiences (Isobel et al., 2017), refers to different types of stressful and traumatic events that occur repeatedly and cumulatively, usually over a period of time, and within specific relationships and contexts (Sar, 2011), which are in most cases (about 80%) perpetrated by parents or other caregivers (U.S. Department of Health & Human Services, 2017): for this reason, it has also been proposed as the expression “attachment trauma” (Isobel et al., 2017).

Even if emotional, physical, and sexual abuse constitutes typical forms of developmental traumatization, neglect constitutes the major form of developmental trauma (Woller et al., 2012; U.S. Department of Health & Human Services, 2017; Witt et al., 2017, 2018). CDT is associated as being the genesis of well-defined clinical pictures such as posttraumatic stress disorder (PTSD; Terock et al., 2016; Williamson et al., 2017), borderline personality disorder (BPD; Ford and Courtois, 2014; Liotti and Farina, 2016; Cattane et al., 2017; de Aquino Ferreira et al., 2018), dissociative disorders (DDs; Amos et al., 2011; Sar et al., 2017), somatoform disorders (SDs; Carlier et al., 2016; Roelofs and Pasman, 2016), eating disorders (EDs; Caslini et al., 2016; Pignatelli et al., 2017; Trottier and MacDonald, 2017), sleep disorders (Kajeepeta et al., 2015), mood disorders (Aas et al., 2016; Jaworska-Andryszewska and Rybakowski, 2018), psychosis (Mayo et al., 2017; Williams et al., 2018), substance-related and addictive disorders (Edalati and Krank, 2016; Konkoly Thege et al., 2017), and obsessive compulsive disorder (OCD; Belli, 2014).

It has been proposed, however, that the typical outcome of cumulative relational trauma is a complex posttraumatic stress disorder (complex PTSD; Herman, 1992a; Sar, 2011; Cloitre et al., 2014; Ford, 2015) characterized by alterations in affect and behavioral regulation, interpersonal problems, dissociative symptoms, and somatizations that should be differentiated from the classical PTSD, according to the Diagnostic and Statistical Manual of Mental Disorders 4th edition, text revision (DSM-IV-TR; American Psychiatric Association, 2000). Furthermore, some scholars proposed “developmental trauma disorder” to describe the clinical presentation in children and adolescents who have been subjected to complex trauma (Van der Kolk, 2005).

Despite empirical support of its existence, specific features of it, and the general agreement on its clinical relevance from experts in the field of psychological trauma, complex PTSD has not been included in the latest edition of the DSM (American Psychiatric Association, 2013) but is present in the International Classification of Disease, Eleventh Revision (ICD-11; World Health Organization, 2018). However, in the DSM-5, a dissociative subtype of PTSD (D-PTSD) was included, defined by symptoms of depersonalization and derealization in addition to symptoms of PTSD (American Psychiatric Association, 2013). A growing amount of clinical and neuroscientific evidence seems to demonstrate significant differences between the PTSD and its dissociative subtype, indicating the latter as typical related to severe and prolonged trauma experiences, early childhood trauma histories, and distinctive treatment needs (Choi et al., 2017; Van Huijstee and Vermetten, 2018). According to recent studies, this dissociative subgroup represented from 12 to 50% of individuals with PTSD and was characterized by more severe PTSD symptoms combined with marked elevations on items assessing flashbacks, derealization, and depersonalization with an higher number of comorbid axis I disorders, avoidant and BPD behaviors, and a more significant history of childhood abuse and neglect (Choi et al., 2017; Van Huijstee and Vermetten, 2018). Moreover, in the DSM-5, among the Associated Features Supporting Diagnosis of PTSD is specified that “following prolonged, repeated, and severe traumatic events (e.g., childhood abuse, torture), the individual may additionally experience difficulties in regulating emotions or maintaining stable interpersonal relationships, or dissociative symptoms” (American Psychiatric Association, 2013). However, some authors raised specific criticism about the D-PTSD. Sar (2017) recently reminded that dissociative phenomena are characterized by not only avoidance symptoms such as detachment symptoms but also positive symptoms such as intrusion. Similarly, Dorahy and Van der Hart (2015) claimed that dissociative symptoms are not restricted to depersonalization and derealization but included negative symptoms such as amnesia, emotional numbing or positive-like flashbacks, trauma-related physical pain, or the passive influence of other dissociative part flashbacks. Authors also argued that the conception of a dissociative subtype leads to a false dichotomy because dissociative symptoms are present even in those individuals with non-dissociative PTSD. Indeed, several studies demonstrated that the dissociative symptoms are present in all PTSD patients along a continuum rather than being a categorical variable characterizing a small subset of individuals (Van Huijstee and Vermetten, 2018). As it will be extensively discussed in this article, we further address it as arguable that the presence of dissociative symptoms could be considered a psychopathological dimension affecting all psychiatric disorders characterizing patient subgroups with histories of traumatic development and low response to treatment (Farina and Liotti, 2013). In any case, as noted by Choi et al. (2017) “this change in the DSM-5 reflects the growing evidence base demonstrating that trauma, dissociation, and posttraumatic stress frequently co-occur in survivors of maltreatment” and, we add, cumulative developmental trauma.

A critical reason for paying heed to the clinical outcome of cumulative developmental trauma, notwithstanding the failed inclusion of complex PTSD among the DSM-5 disorders and the controversy on D-PTSD, is the association between developmental trauma with almost all psychiatric disorders, complicating their clinical pictures with a complex constellation of symptoms that worsen the prognosis (Farina and Imperatori, 2017). Indeed, an increasing number of studies and clinical observations show that an history of cumulative childhood trauma and comorbidity with dissociative symptoms, complex PTSD, or any other traumatic spectrum disorders (e.g., PTSD, BPD, DDs), regardless to a specific diagnostic category, worsens the prognosis of DSM disorders and generates treatment difficulties that require specific knowledge and training for clinicians (Read et al., 2005; Fontenelle et al., 2007; Johnstone et al., 2009; Spitzer et al., 2009; Cloitre et al., 2010; Newman et al., 2010; Szajnberg et al., 2010; McCrory et al., 2017).

The clinical complexity of the sequelae of developmental trauma may contribute to a relative neglect, in the professional community of psychiatrists and psychotherapists, of the proper diagnoses and of the need to implement specific treatment strategies because of some controversial issues that will be discussed in this article.

Many scholars believe that the multiple and complex symptoms caused by childhood trauma have a common pathogenic basis in dissociative processes caused by traumatic attachment relationships with the caregivers during the early years of life and confirmed by subsequent traumas (Liotti, 2004; Van der Hart et al., 2006; Gleiser et al., 2008; Cloitre et al., 2009; Amos et al., 2011; Liotti and Farina, 2016; Farina and Imperatori, 2017). The clinical and empirical evidence led to the consideration that these dissociative pathogenic processes of attachment trauma generate dissociative symptoms likely to dominate some clinical pictures (e.g., PTSD, BPD, and DDs). At the same time, dissociation and a personal history of developmental trauma may also surface, in different proportions, in practically all DSM-IV diagnostic categories as an index of the serious condition of a patient and negative treatment outcomes (Farina and Liotti, 2013). For this reason, as will be discussed extensively later, we proposed to define the dimension of psychopathological outcomes linked to developmental trauma as the “traumatic-dissociative” dimension (TDD; Farina and Liotti, 2013; Farina and Imperatori, 2017). The present article aims to review some crucial points about the clinical meaning and treatment strategies of the TDD, the dissociative pathogenic processes characterizing the TDD, as well as of the role of attachment trauma in the TDD.

## Epidemiology of Developmental Trauma: The Hidden Epidemic

Child trauma is considered a crucial public health problem, associated with both significant individual functional impairments (Gilbert et al., 2009; Fegert and Stotzel, 2016) and social consequences (e.g., high financial burden; Fang et al., 2012; Habetha et al., 2012). Epidemiological data about child abuse and neglect provide prevalence rates ranging from 4 to 16% (Gilbert et al., 2009). The inconsistencies among epidemiological evaluations are attributed to differences in definitions of child maltreatment and to difficulties in collecting data (Stoltenborgh et al., 2011).

It has been argued that most studies probably underestimate the real prevalence rates because parents or other adults who are involved (teachers and social workers) under-report maltreatments because of misunderstanding, memory deficits, denial, attempts to avoid legal problems, and shame (Gilbert et al., 2009). In the scientific literature, different forms of child maltreatment are described: physical, sexual, and emotional abuse, and neglect. They may be found separately or, more frequently, can occur in a combination, with emotional and physical neglect seeming to be the most frequent type of child maltreatment reported (Fairbank and Fairbank, 2009; Witt et al., 2017, 2018).

According to the Child Maltreatment report of 2015 by the US Department of Health, about 10% of the general population experiences one or more forms of maltreatment or abuse in childhood, in most cases (over 80%) perpetrated by parents or relatives (U.S. Department of Health & Human Services, 2017). A meta-analysis (Stoltenborgh et al., 2011) focused on child sexual abuse of 9,911,748 individuals showed that the overall prevalence of this kind of childhood trauma was around 18% for women and 7.6% for men. For other types of childhood trauma, prevalence rates of 22.6 and 36.3% were, respectively, reported for physical abuse (Stoltenborgh et al., 2013b) and emotional abuse (Stoltenborgh et al., 2012). Finally, regarding physical and emotional neglect, a comprehensive meta-analysis (Stoltenborgh et al., 2013a) on 13 independent samples with a total of 59,406 participants reported an overall estimated prevalence of 16.3 and 18.4%, respectively, for physical neglect and emotional neglect, with no significant gender differences.

A personal history of abuse or neglect in childhood seems to be a major factor in causing psychiatric disorders (Herman, 1992b; Green et al., 2010; Carr et al., 2013). A large epidemiological study conducted on a sample of 5,692 psychiatric patients showed that approximately 44% of mental disorders with onset in childhood and 30% of those with onset in adulthood are associated with developmental trauma (Green et al., 2010). Similarly, a systematic review (Carr et al., 2013) on 44 articles reported that the subtypes of childhood trauma (especially physical neglect) can predict the development of psychopathology in adults. Specifically, the authors showed that: (1) physical abuse, sexual abuse, and neglect were related to mood disorders and anxiety disorders; (2) emotional abuse was associated with personality disorders and schizophrenia; and (3) physical neglect was significantly related to personality disorders. It is crucial for further studies to explain the separate contribution of different traumatic childhood experiences and adversities for developing psychopathology. For instance, a recent meta-analysis indicated that, although emotional, sexual, and physical abuse, and domestic violence showed strong associations with depressive risk, neglect was found to be the strongest risk factor for developing depression/depressive symptoms, particularly in females (Mandelli et al., 2015).

It is also important to note that repeated clinical observations and an increasing number of controlled studies show that memory of childhood adverse experiences, traumatic-dissociative symptoms, and comorbidity with traumatic spectrum disorders such as DDs, SDs, BPD, or PTSD are predictors of unsatisfactory therapeutic responses and a tendency to relapse. In general, such an association involves a negative prognosis in patients with mood disorders, anxiety disorders, OCD, EDs, schizophrenia, and several personality disorders (Vanderlinden et al., 1993; Michelson et al., 1998; Zanarini et al., 1998; Brady et al., 2000; Resick et al., 2003; Rufer et al., 2006; Vogel et al., 2006; Brewerton, 2007; Spitzer et al., 2007; Ross, 2009; Thase, 2009; La Mela et al., 2010; Lewis et al., 2010; Mandelli et al., 2015; McCrory et al., 2017; Trottier and MacDonald, 2017).

Paradoxically, in many cases, maltreatment should be reported by parents who instead are either perpetrators of abuse or fail to protect the child from it (neglecting parents). Thus, despite the high estimated prevalence of it, abuse and neglect could be hidden in many apparently well-functioning families and remain unexpressed in the victim’s personal experience until the onset of a disorder in adulthood. For this reason, childhood cumulative trauma may be defined a “hidden epidemic” (Lanius et al., 2010b). As described above, one of the reasons for the epidemic diffusion of developmental trauma being so hidden could be the neglect, by some researchers and clinicians, of some forms of developmental trauma that are less evident, such as traumatic attachments (Liotti, 2004; Liotti and Farina, 2016).

## The Problematic Definition of Developmental Trauma and the Role of Attachment Trauma

One of the most challenging problems for researchers and clinicians is the definition of cumulative childhood traumas (Denton et al., 2017), especially those occurring within familial relationships of attachment and that are known to have particularly profound and complex effects on mental health. These types of childhood traumas have been named in the scientific literature with different but partially overlapping terms: early relation trauma, developmental trauma, complex trauma, or attachment trauma (Schore, 2009; Isobel et al., 2017).

Psychological trauma is usually defined as an unbearable and inescapable threatening single or continuative experience in the face of which a person is powerless (Krystal, 1988; Herman, 1992b; Van der Kolk, 1996). It is clear that the traumatizing power of an event depends not only on how it is harmful or threatening but also on how much it overwhelms the individual’s ability to cope. In other words, the capacities to escape or to resist the threatening experience determine the vulnerability to trauma (Herman, 1992b). In light of this, childhood, as state in which the individual is totally dependent on parents, is an intrinsic condition of vulnerability if caregivers are, in addition to actively abusive or threatening, neglectful and also abdicating of their caregiving role (Liotti, 2004; Isobel et al., 2017).

Human beings, as with all mammals and several other species, have an innate inclination to look for care, help, and comfort from a member of the social group in time of danger, loneliness, and physical or mental pain (Lorenz, 1949; Harlow and Zimmerman, 1959; Bowlby, 1969/1982). This lifelong inclination is one of the main systems regulating and driving human interpersonal behavior and intersects with other interpersonal motivational systems (e.g., those involved in the dominant or subordinated role-relationships, peer cooperation, and sexual bonding; Liotti and Gilbert, 2011; Liotti, 2017). According to Bowlby (1969/1982) and his attachment theory (AT), memories of the real interactions between children and their caregivers are stored in a structure of memory and expectation called internal working model (IWM). The IWM influences personality development and affects later relationships, because it shapes an individual’s expectations of responses to his or her requests for care and comfort (Bowlby, 1969/1982). The considerable body of evidence collected from research on AT shows that the relationship with attachment figures (AFs) may change the neuroanatomical structures involved in the functioning of the attachment system and affect the development of the person’s emotional, cognitive, and meta-cognitive skills (Cassidy and Shaver, 2008; Fonagy and Target, 2008; Vrticka and Vuilleumier, 2012; Gander and Buchheim, 2015).

A wealth of evidence of controlled empirical studies showed that attachment relationships during the first year of life become disorganized as a consequence of maltreatment as well as in response to the interaction with a vulnerable, frightened, but not overtly maltreating parent (Schore, 2009; Granqvist et al., 2017). These controlled studies empirically support the hypothesis that a parent’s mental state mediated by either his/her frightened and abdicating or threatening behavior disorganizes the infant’s attachment pattern through severely disturbed intersubjective processes of communicative misattunement that causes the disaggregation of the infant’s developing mental functions (Carlson et al., 2009; Granqvist et al., 2017). The experimentally observed disorganization of the child attachment mode was indicated with the term “disorganized attachment” (DA; Main and Solomon, 1986; Main and Hesse, 1990).

Disorganized infant attachment is more common among maltreated infants but does not necessarily indicate active maltreatment (Granqvist et al., 2017). Nevertheless, the relationship of an infant with a parent who, because of his/her vulnerability, abdicates parental responsibilities (Solomon and George, 2011) and has become in some way neglecting, has been regarded as “early relational trauma” or “attachment trauma,” capable of causing effects similar to those of other forms of developmental trauma (Schore, 2009).

About 25 years ago, Giovanni Liotti hypothesized that the similarities between dissociative phenomena and behaviors in DA children and their parents may be due to the dissociative nature of the intersubjective process leading to the DA (Liotti, 1992). This hypothesis was later supported by longitudinal controlled studies (Ogawa et al., 1997; Dutra et al., 2009) and neuroscientific experiments (Farina et al., 2014).

There is a strong correlation between the disorganization of infant attachment and the caregiver’s unresolved grief or trauma reflected in highly inconsistent, frightened, or overtly threatening behavior toward the child (Liotti, 2009). Thus, parents who, according to the child’s powerful innate inclination toward care seeking and their objective disposition to care, are perceived by the child as a source of nurturing, simultaneously become a source of threat (Granqvist et al., 2017). Therefore, although DA does not necessarily indicate maltreatment (Granqvist et al., 2017), this paradoxical experience (the parent is at the same time the source of, and the solution to, the child’s fear) is capable of disorganizing the child’s mental processes and represents a type of traumatic experiences constituting an inescapable threatening experience in the face of which the child is powerless (Schore, 2009; Liotti, 2017).

Among others, there are at least three main pathogenic processes activated by DA that could lead to traumatic dissociation. First, the long-lasting stressful and powerless experience of DA activates a chronic stress neurobiological response that interferes with brain development (De Bellis and Zisk, 2014; Teicher et al., 2016). Second, the inescapable threatening condition created by the simultaneous and conflicting activation of attachment and survival defense systems triggers an autonomic parasympathetic detachment response (Porges, 2007; Farina et al., 2015). Finally, the simultaneous tendencies to get close to and flee from the parent cannot be assimilated into the same framework of meaning, because a one-year old child’s limited consciousness-memory skills cannot integrate them into a single act of mental synthesis (Liotti, 2004, 2009). Consequently, several contradictory and reciprocally dissociated self-representations are constructed. The multiple dissociated representations of the self and the attachment figure in infant DA are risk factors for impaired development during childhood, of capacities for integration of affective memories, and for emotional regulation (Liotti, 2009, 2017). In accordance with Liotti, many other scholars consider DA psychopathological processes of a traumatic nature, especially if severe, prolonged, and confirmed in the course of child development and adolescence (Schore, 2009; Isobel et al., 2017). For this reason, we overtly decide to define these conditions “attachment trauma” (AT). Furthermore, it is important to underline that compared to those clinical disorders (i.e., D-PTSD, DTD and complex PTSD), DA is not a validated individual-level clinical diagnosis as well as a stable feature of the individual child but is “relationship specific” (Granqvist et al., 2017). In other words, DA is a set of behavioral responses to a particular caregiver in a specific laboratory situation (i.e., the strange situation; Granqvist et al., 2017). Also, for this reason, we believe that in a clinical context, it is preferable to use the expression “attachment trauma” (Isobel et al., 2017).

The pathogenetic model based on AT provides an effective explanatory key for linking early relational trauma, through the mediation of later traumas suffered at the hand of attachment figures, to the psychopathological processes that pave the way to the complex clinical picture of CDT. Neglecting, threatening, or outright abusive parental behavior that follows and confirms the deep misattunement characteristic of infants with AT, for instance, instigates the construction of dysfunctional beliefs and expectations, which are typical symptoms of developmental trauma disorder (DTD) or complex PSTD (Van der Kolk et al., 2005): relational problems, a tendency to revictimization, mistrust of self and others, helplessness, despair, a feeling of permanent damage, profound worthlessness, shame, and guilt. The dysfunctional beliefs and interpersonal perceptions that underpin DTD or complex PTSD, the dysregulation of emotions, and the tendency to dissociate have a common root in the interpersonal dynamics of AT. The appreciation of such a common root facilitates the therapeutic task of understanding and treating the very complex manifestations and the inner and relational experience of patients suffering from complex PTSD, or from syndromes where the classic clinical picture of any DSM disorder is complicated by the aftermath of complex developmental trauma.

## Trauma and Dissociation

Trauma and dissociation are two closely related concepts in psychopathology. This is true not only insofar as the epidemiological evidence of the causal relationship between developmental trauma and dissociative symptoms is concerned, but also as regards prevailing theories of the pathogenic mechanism activated by trauma (Liotti, 2004, 2017; Briere et al., 2005; Van der Hart et al., 2006; Schore, 2009; Sar, 2011, 2017; Farina et al., 2014; Farina and Imperatori, 2017).

After more than a century of debate and empirical studies, the concept of dissociation is still discussed and problematic, being far from a clear and common definition (Van der Hart and Dorahy, 2009).

Among others, one reason for this lack of clarity depends on the different uses of the word. Indeed, the term dissociation in psychopathology is essentially used to define three different, though related, concepts: (1) a diagnostic category, DDs according to the ICD-11 and DSM-5; (2) a group of symptoms such as amnesia or derealization; and (3) some pathogenic processes caused by traumatic experiences interfering with the integration of mental functions, or keeping separately different part of the personality (Waller et al., 1996; Holmes et al., 2005; Howell, 2005; Farina and Liotti, 2013; Liotti and Farina, 2016; Farina and Imperatori, 2017; Schimmenti and Sar, 2019). In the literature and in the debate concerning the conceptualization of dissociation, there is sometimes confusion between the description of the dissociative phenomena and the dissociation as a pathogenic process.

A comprehensive exposition of the major different theories on traumatic dissociation exceeds the aims and limits of the present paper (for an overview see Sar, 2017; Schimmenti and Sar, 2019); nevertheless, we would like to report some elements of the current theorizations about it.

According to the ICD-11, dissociation is described as the “involuntary disruption or discontinuity in the normal integration of one or more of the following: identity, sensations, perceptions, affects, thoughts, memories, control over bodily movements, or behavior” (World Health Organization, 2018). Consistently in the DSM-5, dissociation is defined as the loss of high-order integrative capacities of the human mind, specifically the “disruption of and/or discontinuity in the normal integration of consciousness, memory, identity, emotion, perception, body representation, motor control, and behavior. Dissociative symptoms can potentially disrupt every area of psychological functioning” (American Psychiatric Association, 2013).

It is important to underline that both previous the definitions of dissociation, as many others, focus on dissociation as a disruption in the normal integration of different mental functions; we will return to this issue later.

Cardeña and Carlson (2011) provided a concise definition for dissociation: “An experienced loss of information or control over mental processes that, under normal circumstances, are available to conscious awareness, self-attribution, or control (…) Symptoms are characterized by (a) a loss of continuity in subjective experience with accompanying involuntary and unwanted intrusions into awareness and behavior (so-called positive dissociation); and/or (b) an inability to access information or control mental functions or behaviors, manifested as symptoms such as gaps in awareness, memory, or self-identification, that are normally amenable to such access/control (so-called negative dissociation); and/or (c) a sense of experiential disconnectedness that may include perceptual distortions about the self or the environment (…) Our definition includes the senses of psychological compartmentalization (i.e., lack of integration between psychological processes) and experiential detachment (i.e., a consciousness alteration characterized by a sense of estrangement from self or others).” As noted by the same authors, this definition includes the differentiation between two major forms of dissociative phenomena suggested by Holmes and collaborators: *detachment* and *compartmentalization*. Detachment is defined by the subjective experience of “an altered state of consciousness, characterized by a sense of separation (or detachment) from certain aspects of everyday experience, be it the body (as in out-of-body experiences), the sense of self (as in depersonalization), or the external world (as in derealization)” (Holmes et al., 2005). Compartmentalization is characterized by a partial or complete failure “in the ability to deliberately control processes or actions that would normally be amenable to such control; this definition incorporates conditions characterized by an inability to bring normally accessible information into conscious awareness (e.g. dissociative amnesia), which can also be regarded as a control problem (…). Deficits of this kind cannot be overcome by a simple act of will, but are reversible in principle (…). In each case, the functions that are no longer amenable to deliberate control, and the information associated with them, are said to be compartmentalized” (Holmes et al., 2005). Initial evidence supported the bipartite model of Holmes and colleagues (Vogel et al., 2013; Mazzotti et al., 2016). These two forms of dissociative manifestations appear to be compatible with the two types of dissociative pathogenic processes suggested by Dell and O’Neil (2009) that distinguished: (1) the *faculty dissociation* that implies a disruption in the normal integration of the psychological functioning of a given state of consciousness and (2) the *multiplicity* that implies the presence of more than one center of consciousness or self.

Is it possible to track some elements of this bipartite model of dissociation among some theoretical models of dissociation that have achieved support from some empirical evidence. Liotti’s initial model derived by disorganization of attachment (described above) is a compartmentalization/multiplicity model where the conflict between motivations, and the exposure to simultaneous or rapidly alternating incompatible attachment experiences, leads to the construction of mutually incompatible and incoherent internal working models that cause a fragmented and compartmentalized self-knowledge that compromises the serial organization of self-consciousness. According to Liotti (1992), detachment symptoms are the consequences of the impasse created by the contemporary existence of multiple, incoherent, mutually incompatible states of consciousness. Not very different is the model described by the psychoanalyst Philippe Bromberg who considers the dissociation a solution against the affective/cognitive incoherence: “the disjunction that takes place, not between inharmonious mental contents, but between alien aspects of self-between states that are so discrepant that they cannot coexist in a single state of consciousness without potential destabilization of self continuity” (Bromberg, 1998a).

Another compartmentalization/multiplicity model is the *theory of structural dissociation of the personality* by Van der Hart et al. (2006), which proposes that patients with complex trauma-related disorders are characterized by a division of their personality into different prototypical parts, each with its own psychobiological motivational underpinnings. Similarly, the *parallel-distinct structures model* by Vedat Sar (2017) postulates the predominant role of disturbed mutuality between internal world and external reality in trauma-related dissociation: “I propose that dissociative disruption leads to emergence of ‘parallel-distinct’ mental structures rather than to simple disaggregation.” It is important to note that Sar’s hypothesis of dissociation appears to be an adaptive attempt to the trauma induced self-fragmentation where detachment is protective from pain and compartmentalization is the adaptive solution: “I hypothesize that chronic developmental traumatization leads to a functional re-organization of the mind into enduring ‘parallel-distinct structures’ which operate side by side without being fully integrated with each other in aim, content, and process (…) Although devoted to protection of the individual’s unique psychological aspects (i.e., rather than constituting a mere fragmentation in the face of relational trauma), such division of the internal world may undermine the enduring experience of a coherent self-identity.”

## Dissociation and Disintegrative Traumatic Processes

As we noted before, many conceptualizations of dissociation are based on the concept of *normal integration*: dissociation consists of disruption in the normal integration of different mental functions such as consciousness, sense of self identity, emotive perception and control, behavioral control, body representation, and movements (Nijenhuis and Van der Hart, 2011). In other words, dissociation may be conceptualized as a consequence of pathogenic processes that, in different ways, alter or disrupt the intrinsic property of optimal mental functioning: the capacity of integration (Pessoa, 2017). As Janet stated more than a century ago: “mental health is characterized by a high capacity for integration” (Janet, 1889 page 460). The disintegrative processes lead to a wide range of different psychopathological phenomena depending on which mental function they compromise: from alterations of self-consciousness, such as detachment symptoms (derealization/depersonalization), to the fragmentation of self-experiences, such as compartmentalization symptoms (e.g., amnesia, motor control, and multiple personality), and even to a sudden loss of control on emotions and behavior and vegetative arousal dysregulation (Carlson et al., 2009; Farina and Liotti, 2013; Chou et al., 2018).

The latter psychopathological manifestations, although not considered typical dissociation until recently, are very common in people suffering from trauma-related disorders, such as BPD, PTSD, or complex PTSD, and are supposed to be generated by the disintegrative processes activated by the traumatic experience (Meares, 2012; Liotti and Farina, 2016). Modern psychiatry derived this concept from Janet’s theories, where *désagrégation* involves the disconnection of the normally overlapping and integrated different functional levels of the mind, caused by the violent emotions inherent in traumatic experiences (Ellenberger, 1970; Van der Hart and Dorahy, 2006, 2009).

Following Janet’s ideas, many contemporary trauma scholars believe that the loss of integration involved in the dissociative state should include, in addition to the state of consciousness and sense of self, behavior, emotional and impulse control, sensory experiences, body scheme, and image, ability to consider the person’s own mental states and those of others, consistency in representations of self and other people, and autobiographic narratives (Van der Kolk, 1996; Van der Kolk et al., 1996; Nijenhuis et al., 1998, 2003; Meares, 1999, 2000; Van der Hart et al., 2006; Van der Hart and Dorahy, 2006; Carlson et al., 2009).

Some scholars have also hypothesized that dissociative responses to trauma are based on the activation of the archaic defense system located in the brain stem and are responsible for the typical sequence of freezing, fight-flight, and feigned death (Cantor, 2005; Schore, 2009). That is, besides the overwhelming emotions involved in it, trauma triggers archaic behavioral systems evolved in reptiles, birds, and mammals in order to protect the individual organism in life-threatening situations. The archaic defense system when its activation reaches the extreme response of feigned death (flight when no other form of flight is possible) causes a detachment of the conscious experience from the usual sense of self and of the outside world – that is, the dissociative symptoms of depersonalization and derealization sometimes described as dissociative detachment (Holmes et al., 2005; Brown, 2006). The state of detachment caused by trauma, then, may abruptly interrupt the normal functioning of higher mental systems, prevent the integration of the traumatizing event in the continuum of psychological life, and cause, in the long term, a fragmentation in the person’s consciousness, memory and identity, and body representation (symptoms described as dissociative compartmentalization; Van der Hart et al., 2006; Schore, 2009).

We can infer from these observations that the repeated experience of dissociative detachment in childhood, associated with the interaction with abusing caregivers (or simply scared: see before), may very seriously hamper the ability to integrate traumatic experiences and memories in a coherent representation of self and others. Therefore, together with other pathogenetic mechanisms related to complex trauma, such as stress hormones hampering neurogenesis, synaptic plasticity, myelination, and neural network development (De Bellis and Zisk, 2014; Teicher et al., 2016), dissociative detachment generates failures in affect regulation (supported by the integration between the functions of the limbic system and those of the neocortex) and leads to the fragmentation of mental activities, behavioral strategies, and autobiographic memories, as well as of the sense of self (Carlson et al., 2009; Liotti, 2009; Schore, 2009; Teicher et al., 2010; Braun and Bock, 2011; Meares, 2012).

Consistently, several neuroscientific studies have partially demonstrated that dissociation relates to alterations of widely distributed cortical connectivity networks that underpin higher-order integrative mental functions (Hopper et al., 2002; Lanius et al., 2005, 2018; Wolf et al., 2011; Akiki et al., 2017).

It is interesting to note that in some cases dynamic network dysfunctions become evident only after the activation of attachment memories. Farina et al. (2014) found a disruption of cortical connectivity networks in dissociative patients after the recall of traumatic attachment memories by Adult Attachment Interview. Furthermore, in a more recent study (Farina et al., submitted), connectivity disturbances were observed in a population of people exposed to severe dysfunctional parenting exclusively after the activation of early attachment memories. Therefore, it is possible to hypothesize that, in individuals with early adverse relational experiences and attachment trauma, the structural connectivity deficit becomes functionally evident and clinically symptomatic when the system is overloaded by affective and cognitive attachment-related stimuli.

## Are Dissociation and Disintegration the Same Process?

As just described in the previous section, there is a broad consensus among many authors, some of us included, on conceiving dissociation as a lack of integration of different high-order mental functions (Meares, 2012; Liotti and Farina, 2016; Nijenhuis, in press). So, should we consider dissociation and dis-integration synonyms? Are dissociation and dis-integration the same process with different names? As Meares and Barral (2019) recently stated: “It should be noted that the disintegration, that is, désagrégation, is distinguished from the ‘dissociation.’”

Clinical observation and scientific reports led us to hypothesize that dis-integrative effects of trauma-related experiences and dissociation could be two different, but highly correlated, processes. Is it possible to consider that overwhelming emotions and archaic defense system activated by traumatizing events or their following memories affect the structural and dynamic connectivity hindering the normal integration of different high-level mental functions such as consciousness, continuity of self-identity, emotive perception and control, behavioral control, body representation and movements, and mentalization (Sar, 2017; Schimmenti, 2017). This process could be sustained and continued by the long-lasting hampering effect of stress hormones on integrative neuro-structures.

Differently, the dissociation could be considered the integrative failure and the subsequent re-composition of the system’s constituting elements in a more separated way (Van der Hart et al., 2006; Nijenhuis, in press), or, as hypothesized by Sar, the dis-integrative process should be considered the *disruption* mechanism, whereas, instead, the dissociative process consists of the “functional re-organization of the mind into enduring parallel-distinct structures.”

To give an example we can consider the detachment consciousness symptoms (e.g., depersonalization and derealization), the sudden emotive dysregulation, and the abrupt decline of metacognitive monitoring as effects of dis-integration. Conversely, the compartmentalization symptoms (Holmes et al., 2005) such as amnesia, multiple personality, conversion disorders, fragmented sense of self, implicit relational traumatic memories, and long-lasting somatizations or alexithymia can be viewed as products of dissociative process (Schimmenti, 2017). Along with Van der Hart et al. (2006) and Sar (2017), we can consider that the to-become-dissociated system includes dispositions that increase the probability that the parts of the personality become recomposed in a particular way where they are more autonomous and separated.

It is possible to hypothesize that the predisposition to either dis-integration or dissociation is due to a combination of innate temperamental factors (genetically and epigenetically determined) and early experiences, such as infant attachment disorganization, that circularly influence each other (Liotti and Liotti, 2019). Furthermore, a characteristic of the severely disaggregating power of complex (cumulative) trauma is the difficulty or impossibility to ascribe a unified and coherent meaning to it that unfortunately contributes to the dissociative process. The memory of extreme traumatic experiences cannot be included in the orderly system of memories and cannot be integrated with the other information and meanings normally available to a person that constitute his/her identity and sense of self, (Pierre Janet aptly called this inclusion “personal synthesis”; Van der Hart and Dorahy, 2006). The fragmentation of memories and meanings expressing this failure of personal synthesis is reflected, as mentioned above, in the typical symptoms of dissociative compartmentalization: amnesia, flashbacks, and non-integrated ego states, up to the extreme of the dissociative parts of the personality typical of dissociative identity disorder (Van der Hart et al., 2006).

Psychological functions charged with extreme affects, when they are dissociated from the memory of the originating events, could circularly cause the dis-integrative process: sudden and intense emotions manifested in a chaotic and incongruous form, such as unmanageable fears, anxiety, and unjustified bouts of anger (Carlson et al., 2009). The dissociation of psychological functions consequent to trauma may even separate the mental representations of the body from the awareness of the emotions, causing alexithymia and several somatoform symptoms such as motor paralyses, impairment of the sensations, painful syndromes without organic basis, and dysmorphophobias (Sar et al., 2004; Liotti and Prunetti, 2010; Farina et al., 2011).

Dis-integrative and dissociative processes combined together, and with attempts to adapt to threatening and incoherent caregivers during development, also lead to negative self-beliefs and interpersonal problems that are among core symptoms of DTD and complex PTSD (Carlson et al., 2009; Cloitre et al., 2011).

Emerging literature suggests that dis-integrative and dissociative processes would benefit from different therapeutic strategies (Liotti and Farina, 2016; Sar, 2017).

## The Limits of PTSD in Describing Childhood Trauma: Complex PTSD and Other Diagnoses for Developmental Trauma

The clinical picture originating from traumatic developmental experiences features a variety and complexity of symptoms that, it has been argued, cannot be described by the DSM diagnostic criteria for PTSD (Classen et al., 2006; Ford and Courtois, 2009; Chu, 2010; Ford, 2015). It has been argued that these symptom clusters are not suitable to diagnose the adult sequels of developmental trauma (Cloitre et al., 2010). Being designed to describe psychopathological reactions to one, or a small number, of traumatic events within a limited timeframe, this category cannot adequately represent the complex responses to traumatic experiences that, besides being repeated and taking place over a long time span, have very special interpersonal features (e.g., they are inflicted by caregivers) and impinge on personality development (Cloitre et al., 2009; Ford and Courtois, 2009). A multicenter study involving approximately 1,700 children exposed to traumatic events showed that 78% of them suffered multiple and prolonged traumatic experiences and that only 25% of them met the diagnostic criteria for PTSD (Spinazzola et al., 2005).

When a diagnostic system does not include diagnoses capable of capturing the symptoms of a vast population of disturbed persons, these symptoms are inevitably ascribed to other disorders and these patients will be misdiagnosed. An epidemiological study showed that, in 79% of the cases, the diagnosis of PTSD is associated with another axis I diagnosis of the DSM, and in 33% of the cases a diagnosis of personality disorder (Kessler et al., 1995). These rates are comparable to those found in other studies (see, for example, Pagura et al., 2010; Dorrington et al., 2014).

This means that, paradoxically, approximately eight times out of 10, the diagnosis of PTSD is not sufficient to describe the complex suffering consequent to trauma, especially cumulative developmental trauma, and this leads clinicians to resort, sometimes misleadingly, to double diagnoses and comorbidity (Maj, 2005). Repeated, cumulative traumas during the developmental years imply that children have to adapt to expectations of repeating very painful and inescapable experiences and often to the fact that the source of threat is in the same time the source of protection (attachment trauma; Liotti and Farina, 2016).

In order to move beyond the descriptive limits of PTSD and provide specialists with a diagnostic category for prolonged traumas, especially focusing on their interpersonal features, Judith Herman (1992a,b) proposed to include in the DSM-IV the new diagnosis of complex PTSD. First empirical data supporting Herman’s clinical observations came from the DSM-IV field trial for PTSD. The study of a large clinical population led to the conclusion that interpersonal cumulative trauma (defined as complex trauma) produced a different clinical picture than that described for PTSD following the single traumatic events (Van der Kolk et al., 1996; Pelcovitz et al., 1997). The researchers (Van der Kolk et al., 1996, 2005; Roth et al., 1997; Zucker et al., 2006; Cloitre et al., 2011) found that the main differences between the symptoms generated by cumulative trauma and those caused by single traumatic events were a pervasive alteration of affect regulation, different types of somatizations (such as psychogenic pain symptoms, somatoform, and conversion symptoms), state-like dissociation (such as depersonalization and derealization), and more enduring dissociative disturbances of self-identity (e.g., amnesia, ego state fragmentation, and dissociative identity disorder).

Despite the evidence provided by the experimental data, the DSM-IV commission rejected the new diagnosis of complex PTSD proposed by Herman and later reformulated by Van der Kolk and his collaborators as disorder of extreme stress not otherwise specified (DESNOS; Pelcovitz et al., 1997; Van der Kolk et al., 2005). Diagnostic criteria proposed for DESNOS regard the following areas: (1) alterations of affect regulation and impulse control; (2) dissociative symptoms; (3) somatoform symptoms; (4) self-identity disturbances; (5) alterations in the perception of the perpetrator; (6) relational problems; and (7) alterations of personal meanings (such as helplessness, feeling permanently damaged, ashamed, despair, and distrust).

In an expert opinion survey carried out by the Complex PTSD Task Force of International Society for Traumatic Stress Studies (ISTSS), it emerged that affect dysregulation, relationship disturbances, and disturbances in a system of meaning are endorsed by specialists as core symptoms of complex PTSD that are always present (Cloitre et al., 2011).

In recent years, experimental evidence of the existence of a specific nosography of developmental trauma increased (Chu, 2010; Ford and Courtois, 2014; Ford, 2015). Dorahy et al. (2009), in a sample of 81 outpatients with a Belfast-Troubles trauma history, reported that the diagnosis of DESNOS was related to childhood trauma exposure, neglect, and interpersonal difficulties. Interestingly, Cloitre et al. (2009) explored the relationship between symptoms complexity and type of trauma in 582 adults and 152 children. They concluded that “exposure to multiple and repeated forms of maltreatment and trauma in childhood can lead to outcomes that are not simply more severe than the sequelae of single incident trauma, but are qualitatively different in their tendency to affect multiple affective and interpersonal domains” (Cloitre et al., 2009). Despite these data, empirical evidence is still limited, and a debate is still open as to whether the DESNOS should be considered a severe form of PTSD or a separate diagnosis (Dorahy et al., 2009; Cloitre et al., 2010).

Another controversy concerns the overlapping of BPD and complex PTSD (Lewis and Grenyer, 2009; Ford and Courtois, 2014; Sar, 2017), which some would like to resolve by adding the diagnosis of posttraumatic personality disorder (Classen et al., 2006). Furthermore, Lanius et al. (2010a) suggested to add a “dissociative” subtype of PTSD for “chronic trauma of psychological, sexual and physical nature, including neglect such as parents’ psychological unavailability.” Despite these proposals, the last edition of the DMS (American Psychiatric Association, 2013) only added to PTSD diagnosis the specification “with dissociative symptoms” that should be reported when depersonalization and derealization symptoms are present.

Given the lack of a single, officially acknowledged diagnosis for the disorder originated by cumulative developmental traumatic experiences, in the last years, we proposed to introduce a dimensional psychopathology perspective in order to better understand the role of attachment disorganization and the activation of dis-integrative and dissociative processes in the clinical course and their therapeutic implications (Farina and Liotti, 2013; Farina and Imperatori, 2017). In agreement with many others, as evidenced by an even cursory reading of the literature on complex trauma (Lanius et al., 2010a), we believe that a correct identification of the multiform manifestations of trauma-related dissociative pathogenic process has important therapeutic implications likely to improve the prognosis of the patient regardless the specific diagnostic category. Clinicians must be prepared to recognize the signs of the traumatic-dissociative dimension in order to implement the specific techniques capable of resolving the problems it creates.

## Traumatic-Dissociative Dimension: Some Reflections on its Usefulness

The reason for the rejection of complex PTSD by APA was explained by Herman (2009) herself: it features symptoms quite typical of dissociative, somatoform, and personality disorders and therefore does not seem properly ascribable to anxiety disorders (as PTSD was in DSM-IV). Moreover, it could be impossible to decide whether to include it among the dissociative, the somatoform, or the personality disorders (Herman, 2009). It is certainly understandable that a disorder defined on the basis of an etio-pathogenic mechanism may cause allocation problems in a category-based diagnostic system such as the DSM (Chu, 2010). Furthermore, it is just as understandable that the multiple, protean, and changeable symptoms caused by complex trauma (albeit they allegedly share a common ground in the TDD) may lead clinicians to multiply the diagnoses and use the expedient of comorbidity (Maj, 2005). The reliability of the DSM and ICD systems on capture trauma and dissociative-related disorders has been greatly increased (for instance, through the introduction of complex PTSD in ICD-11), improving diagnostic agreement among clinicians and providing researchers with rigorous diagnostic standards. Nevertheless, the use of diagnostic categories of these classification systems risks producing an impoverished view of psychopathology and less clinical sensitivity and precision (Picardi et al., 2017).

To take into account the concept of the psychopathological dimension is a way to move beyond the descriptive limits of current international categorical diagnostic systems in describing the consequences of developmental trauma: “a dimensional perspective may allow more precision in understanding the posttraumatic symptoms” (Briere and Spinazzola, 2005).

Since childhood trauma interferes with normal development of different mental functions, the heterogeneity of its clinical outcome is not surprising. Dis-integrative and dissociative pathogenic processes activated by developmental trauma lead to various alterations in self-identity and self-regulatory capacities and relational problems that cause the multiform complexity of clinical outcomes. We hypothesize that when this complexity is viewed as based on a single psychopathological dimension, it can characterize either a well-defined clinical picture, such as DTD and complex PTSD (Van der Hart et al., 2005), or slightly atypical forms of different DSM disorders – in the latter case worsening their prognosis (McCrory et al., 2017). Regardless of the DSM diagnosis, patients with developmental trauma share some psychopathological traits and respond worse to conventional therapies, which have been the finding of several studies (Gleiser et al., 2008; Cloitre et al., 2010, 2011; Bausch et al., 2017; McCrory et al., 2017).

Comorbidities with dissociative symptoms, emotive and behavioral dysregulation, and relational difficulties affecting the therapeutic relationship and alliance seem to characterize patient subgroups with histories of traumatic development and, crucially, with low responses to treatment (Farina and Liotti, 2013; McCrory et al., 2017). Sar (2017) recently stated: “Dissociation is a constant feature of post-traumatic conditions independent of the main psychiatric diagnosis.” An increasing amount of empirical controlled data supporting this hypothesis are available for several mental disorders (Schilling et al., 2015) including psychosis (Misiak and Frydecka, 2016), mood disorders (McBride et al., 2006; Harkness et al., 2012; Cakir et al., 2016; Prasko et al., 2016), SDs (Jepsen et al., 2014), OCD (Rufer et al., 2006; Semiz et al., 2014), EDs (Waller, 1997; Carter et al., 2006; Trottier and MacDonald, 2017), and anxiety disorders (Michelson et al., 1998; Prasko et al., 2016).

To give only an example, Bausch et al. (2017) recently specified that “Childhood maltreatment has been identified as predictive for a poor longitudinal depression course, an increased burden of the illness, and poor treatment response.” Moreover, Jobst et al. (2016), in a review on chronic forms of major depressive disorder (MMD), concluded that the development and persistence of chronic depression are often related to an history of multiple childhood traumas and abuse such as significantly poorer parental care, emotional neglect, and emotional abuse that were found to be the most common subtypes of interpersonal trauma: “the rate of childhood trauma among chronic depression patients is estimated to be up to 80% (…) and severity of childhood trauma has been suggested to be associated with chronicity of MMD.”

This TTD, when associated with other disorders, has been hypothesized and partially demonstrated to worsen prognosis and lead to specific therapeutic difficulties.

## Treatment Difficulties and Therapeutic Principles for the Traumatic-Dissociative Dimension

As just described, clinical and experimental evidence suggest that, regardless of the DSM or ICD diagnosis, patients with developmental trauma histories respond worse to conventional therapies. The presence of signs and symptoms of TTD concur in complicating treatment, both when they are grouped in a DD or in complex PTSD and when they are associated with another diagnosis. Traumatic attachment and other forms of early relational trauma lead to a sense of mistrust and powerlessness, the traumatic memories related to the care interaction determine the attachment phobia, and all of these are obstacles to the construction of the therapeutic alliance necessary for any form of therapy (Van der Hart et al., 2006; Liotti et al., 2008; Kinsler et al., 2009; Liotti, 2017).

Distortions in consciousness continuity and memory, a fragmented sense of self, difficulty in arousal regulation, affective and behavioral dysregulation, other forms of somatization hinder or prevent effective use of therapeutic techniques and strategies developed for the treatment of several disorders when they are not associated with trauma (Farina and Liotti, 2013). The treatment of patients with TDD is complex and must rest on a multi-phase program with some important goals and steps (Courtois and Ford, 2009; Cloitre et al., 2011), as described below.

## Construction of Therapeutic Alliance, of Safety and Security in the Therapeutic Relationship

The main barrier in treating developmental trauma disorders and traumatic-dissociative symptoms, as it could have been inferred by the reading of previous paragraphs, is the difficulty in the construction of that atmosphere of mutual trust and cooperation, typical of effective professional care relationships, called therapeutic alliance (Safran and Muran, 2000). Therapeutic alliance is the basis upon which clinicians of all traditions and persuasion who are acknowledged experts in the therapy of developmental trauma disorders build and maintain the whole course of treatment, regardless of whether it is a psychotherapy or a pharmacological treatment (Allen et al., 2008). Usually a good therapeutic alliance is the starting condition to initiate treatment, for TDD patients this is almost invariably the first and most complex goal, although it is not always attainable in full and often requires months (and sometimes years) of therapeutic work to be achieved (Courtois et al., 2009; Kinsler et al., 2009; Liotti and Farina, 2016). The difficulty in the construction of the therapeutic alliance, which greatly complicates the therapy of TDD patients, is due to the interpersonal dynamics consequent to their traumatic relational experiences during developmental years, through the mediation of reactions triggered by disorganized attachment memories, pathogenic beliefs, and negative expectations (Fosha, 2003; Liotti, 2004; Liotti and Farina, 2016). All these reasons and their diverse and enmeshed interconnections make it difficult, if not impossible, to create a stable therapeutic alliance and avoid empathic failures (Bromberg, 2008; Stern, 2008). Thus, managing the unavoidable empathic failures and repairing the recurrent ruptures in the therapeutic alliance are crucial aspects of therapy (Sommerfeld et al., 2008). When interpersonal dynamics, linked to memories of complex developmental trauma and early traumatic attachment, take the stage in the clinical exchange, the client’s emotional experience inevitably affects the therapist’s, and this may cause serious ruptures of the therapeutic alliance (Liotti et al., 2008; Stern, 2008).

The role of the psychotherapist (and one of the therapy goals) is to understand and openly share what happens with the client, turning a possible empathic failure into a precious therapeutic opportunity (Sommerfeld et al., 2008; Meares, 2012). In fact, distress modulation in the therapeutic relationship exposes clients to an emotional and relational experience allowing them to review and correct the pathogenic beliefs related to traumatic childhood relations (Bromberg, 1998b; Fosha, 2003; Stern, 2008; Courtois et al., 2009; Kinsler et al., 2009; Meares, 2012). The accelerated experiential-dynamic psychotherapy (AEDP) is just one example of psychotherapy informed by attachment theory that focuses on the therapeutic relationship (Gleiser et al., 2008; Fosha et al., 2009). AEDP aims to promote patients’ sense of safety and to regulate his/her affective state by the interpersonal emotional attunements activated in the attachment relationship between patient and therapist.

The creation of a good therapeutic alliance in DTD treatment, and the repair of its ruptures, also paves the road to the investigation of the relational dynamics underlying the symptoms, and to the possible implementation of specific techniques aimed at changing them (Spermon et al., 2010). The construction and management of the therapeutic alliance require clinicians, in addition to other specific skills, to take attitudes likely to be effective in any treatment but essential for DTD: shared formulation of therapeutic goals and tasks, sharing and respecting the mutual therapeutic roles, safeguarding setting rules, and a generally open, frank, honest, welcoming, calm, kind, and respectful attitude of sincere interest and empathic attunement to the client’s needs. This is what all human beings want to receive in a helping relationship, and this is what clients with early relational traumas did not receive during their personality development, to such an extent that they are unable, during their adulthood, to welcome and receive it when it is finally offered to them (Courtois and Ford, 2009; Courtois et al., 2009; Kinsler et al., 2009). The therapists’ attitudes favoring authenticity, sharing, and mutual respect, in order to be acknowledged and properly received by patients with severe developmental and relational trauma, must be integrated with specific skills: the ability to recognize and predict maladaptive interpersonal dynamics, the careful use of emotion validation interventions, and the capacity to monitor and foster the patient’s mentalization skills (Dimaggio et al., 2010). The creation of a good therapeutic alliance and the prompt repair of its ruptures, together with an active engagement of the therapist in alleviating or at least stabilizing the patients’ most disabling symptoms (dissociative detachment symptoms, impulsive actions and at-risk behaviors, dysregulated rage, anxiety and sadness, and repetition of abusive relations) allow for the patients’ experience of the therapeutic relationship to be as a reasonably safe haven (Fosha, 2003). These goals of the first stage, which involve corrective relational experiences with respect to the patients’ early experience of DA relationships, are a pre-condition for the “trauma work” that characterizes the subsequent phases of the treatment (Cloitre et al., 2011).

### Major Tasks and Specific Therapeutic Techniques for the Treatment of TDD Symptoms

After working at the construction and maintenance of the therapeutic alliance, the other specific tasks for the treatment of clients with traumatic developmental experiences aim at arousal and emotion regulation, stabilization of detached dissociative states and somatoform symptoms, exploration of traumatic memories, resolution of pathogenic beliefs, desensitization of the phobias of inner mental states, and, finally, integration of dissociative parts of the personality (Van der Hart et al., 2006; Courtois et al., 2009; Bryant, 2010; Van der Kolk, 2014). For example, patients with complex trauma-related disorders are often characterized by a division of their personality into different prototypical parts, each of which has dysfunctional behaviors maintaining dissociation (Steele et al., 2001; Van der Hart et al., 2006). In these cases, specific therapeutic protocols, such as the phase-oriented treatment, are recommended in order to help patients to gradually develop function mental and behavioral strategies promoting the integration (for details, see Steele et al., 2005).

The expert opinion survey of the ISTSS Complex PTSD Task Force indicates that there is a major agreement on the use of multiple types of interventions tailored to the most prominent symptoms. In particular, survey results indicate that emotion regulation, education about trauma, and cognitive restructuring were designated as highly effective first-line interventions (Cloitre et al., 2011). In order to pursue these goals successfully, therapeutic techniques specifically designed for the traumatic-dissociative dimension must be mastered, alone, or in combination with pharmacological therapies. A detailed description of these techniques or of the integration modes used in different treatments exceeds the scope of this article; we shall therefore just name some of them and make reference to the relevant literature for a more complete discussion (Chu, 1998; Dworkin, 2005; Classen et al., 2006; Ogden et al., 2006; Van der Hart et al., 2006; Courtois et al., 2009; Spermon et al., 2010; Cloitre et al., 2011; Howell, 2011; Van der Kolk, 2014; Frewen and Lanius, 2015; Ogden and Fisher, 2015; Fisher, 2017).

One of the specific tasks of the TDD psychotherapy is the treatment of the dissociative symptoms of detachment. For this purpose, therapeutic tools and techniques were developed. They are designed to ground dissociative patients to the concrete (internal and external) reality and to orient them in the here and now, offsetting detached dissociative states through the exploration and enhancement of intense sensory stimulation from the outside world (lights, sounds, tactile stimuli) or from the body (focusing on proprioception of intense body sensations) (Chu, 1998; Van der Kolk, 2014; Frewen and Lanius, 2015; Ogden and Fisher, 2015; Fisher, 2017).

Another essential and specific task in the treatment of complex trauma is the work on traumatic memories. Working on traumatic memories requires specific techniques and a general attitude of extreme caution, bearing in mind that the goal of therapy is not the reconstruction of truth, but rather validation of the client’s painful experience and integration of those dissociated memories that produce disabling symptoms. To this end, memory recollection should not be forced, rather awareness of the false memory problem should be borne in mind (Brown et al., 1998; Loftus, 2003; Dalenberg and Palesh, 2010), and therapeutic procedures such as eye movement desensitization and reprocessing (EMDR) should be used. EMDR is one of the most widely used evidence-based therapeutic techniques for the treatment of traumatic memories (Korn and Leeds, 2002; Shapiro, 2002; Dworkin, 2005; Van der Kolk et al., 2007; Schubert and Lee, 2009; Nardo et al., 2010). It is postulated that EMDR promotes the integration of traumatic memories and the regulation of the associated emotional states and pathogenic negative beliefs, increasing inter-hemispheric communication through the eye movements or alternate stimulations of left and right parts of the body (e.g., alternate tapping on the client’s right and left hand; Propper et al., 2007; Schubert and Lee, 2009).

Sensorimotor psychotherapy (SMP) was successfully developed to treat the arousal dysregulation and the somatic and preverbal consequences of traumatic experiences (Ogden and Fisher, 2015; Fisher, 2017). Its main goal is to help clients regulate autonomic functions altered by developmental trauma, modify somatoform symptoms, and change negative beliefs about the body. As stated by Fosha, “Therapies dealing with disorders that are fundamentally emotional in nature need to be able to reliably access sensory, motoric, and somatic experiences to engage them in a dyadic process of affect regulation and eventual transformation. This requires a bottom-up processing approach of experiential therapies, rather than the top-down approach of most cognitive and insight-focused therapies” (Fosha, 2003). This goal in SMP is pursued first by carefully tracking and acknowledging the sensorimotor patterns in which these dysfunctions are reflected: postures, feelings of muscular tension, specific movements, and body experiences. Thereafter clients are encouraged, during the session, to associate these patterns of bodily experiences with emotional states, thoughts, and traumatic memories. Thus, sensorimotor therapy helps clients recover the awareness and mastery of their body experiences and enables them to stop avoidance caused by the negative quality of the somatic aspect of traumatic experience. It progressively promotes the ability to modulate the somatic activation caused by dysregulated emotions; fosters modification of the pathogenic beliefs of worthlessness, guilt, and shame related to the body; and improves clients’ relational skills. Lastly, body attunement techniques between therapist and patient, in addition to improving control over the body and mitigating somatoform symptoms, also strengthen the therapeutic alliance (Fisher and Ogden, 2009).

It is worth remembering that both the therapy general principles and some therapeutic techniques developed to treat complex trauma and dissociation can be successfully applied to group family and couple therapies, and that particular forms of trauma and dissociation therapies have been specifically devised for application in group contexts (Courtois and Ford, 2009; Astrachan et al., 2010).

Finally, use of medications in clients with developmental traumatic experiences needs to be briefly mentioned. Such treatments are especially useful in the first stage of therapy, stabilizing the most disabling symptoms. Though pharmacological therapies alone cannot control the clinical picture, their use is recommended to manage some specific symptom clusters (such as emotional dysregulation and poor impulse control, irritability, and intrusive symptoms) or to treat the frequent comorbid disorders such as mood or anxiety disorders (Stein et al., 2006; Opler et al., 2009). Use of medications in the TDD does not differ in terms of modes and dosage from other clinical areas, but it is worth remembering that specific cautions should be taken, in order to avoid collusion between possible unwanted effects of medications and the tendency to distrust self and others, typical of these clients – a collusion that could be detrimental to the therapeutic alliance. This is why the clinician in charge of the therapeutic plan management should work jointly with the colleague prescribing the medications.

## Conclusion

The complexity of TDD treatment often requires simultaneous use of different types of intervention, as in the abovementioned case of psychotherapy and pharmacological therapy, or in the case of association between different types of psychotherapy (cognitive-behavioral or psychodynamic interventions associated with group, couple, or family therapy), or between techniques stemming from different approaches to psychotherapy such as EMDR or SMP (Cloitre et al., 2011). As Bryant (2010) recently pointed out, the clinical and therapeutic complexity of developmental trauma and of the traumatic-dissociative dimension requires specific training to enable mental health professionals to recognize the signs of these developments and to implement specific strategies for the treatment of these disorders. In order to overcome the difficulties created by childhood attachment trauma and dissociation, these specific strategies must be associated with more conventional therapies.

It has been argued in guidelines for the treatment of developmental trauma spectrum disorders that the management of multiple interventions could profitably be entrusted to different therapists working jointly (Liotti et al., 2008). This allows clients to have several attachment figures, working as one team (Bateman and Fonagy, 2004) to rely on within the therapy’s overall plan. It also allows therapists to defuse the dynamics triggered by the DA and share the interpersonal difficulties typical of these disorders as well as the management of their complex clinical pictures (Liotti et al., 2008; Liotti and Farina, 2016). The consequence of this line of reasoning is that the treatment of TDD and developmental trauma spectrum disorders such as complex PTSD or BPD may well become a privileged ground for psychotherapy integration and for the integration between biological psychiatric thinking and psychotherapeutic approaches to treatment: not a secondary reason for exploring these trauma-related disorders, and not a secondary reason among those that prompted us to write this article.

## Author Contributions

BF, ML, and CI contributed to the study design and literature searches. BF and CI wrote the manuscript. BF contributed to supervision. ML edited the manuscript.

### Conflict of Interest Statement

The authors declare that the research was conducted in the absence of any commercial or financial relationships that could be construed as a potential conflict of interest.

## References

[ref1] Aas M. Henry C. Andreassen O. A. Bellivier F. Melle I. Etain B. (2016). The role of childhood trauma in bipolar disorders. Int. J. Bipolar Disord. 4:2. 10.1186/s40345-015-0042-0 26763504 PMC4712184

[ref2] Akiki T. J. Averill C. L. Abdallah C. G. (2017). A network-based neurobiological model of PTSD: evidence from structural and functional neuroimaging studies. Curr. Psychiatry Rep. 19:81. 10.1007/s11920-017-0840-4 28924828 PMC5960989

[ref3] Allen J. G. Fonagy P. Bateman A. W. (2008). Mentalizing in clinical practice. (Washington, DC/London: American Psychiatric Publishing Inc).

[ref4] American Psychiatric Association (2000). Diagnostic and statistical manual of mental disorders IV-TR. (Washington, DC: American Psychiatric Association).

[ref5] American Psychiatric Association (2013). Diagnostic and statistical manual of mental disorders – DSM-5. (Arlington: American Psychiatric Publishing).

[ref6] Amos J. Furber G. Segal L. (2011). Understanding maltreating mothers: a synthesis of relational trauma, attachment disorganization, structural dissociation of the personality, and experiential avoidance. J. Trauma Dissociation 12, 495–509. 10.1080/15299732.2011.593259, PMID: 21967177

[ref7] Astrachan T. Bernardes C. Herman J. L. (2010). “Part 6 synopsis” in The impact of early life trauma on health and disease: The hidden epidemic. eds. Lanius R. A. Vermetten E. Pain C. (New York: Cambridge University Press), 295–299.

[ref8] Bateman A. W. Fonagy P. (2004). Mentalization-based treatment of BPD. J. Personal. Disord. 18, 36–51. 10.1521/pedi.18.1.36.32772, PMID: 15061343

[ref9] Bausch P. Fangmeier T. Zobel I. Schoepf D. Drost S. Schnell K.. (2017). The impact of childhood maltreatment on the differential efficacy of CBASP versus escitalopram in patients with chronic depression: a secondary analysis. Clin. Psychol. Psychother. 24, 1155–1162. 10.1002/cpp.2081, PMID: 28326653

[ref10] Belli H. (2014). Dissociative symptoms and dissociative disorders comorbidity in obsessive compulsive disorder: symptom screening, diagnostic tools and reflections on treatment. World J. Clin. Cases 2, 327–331. 10.12998/wjcc.v2.i8.327, PMID: 25133142 PMC4133421

[ref11] Bowlby J. (1969/1982). Attachment and loss. (London: Hogarth Press).

[ref12] Brady K. T. Killeen T. K. Brewerton T. Lucerini S. (2000). Comorbidity of psychiatric disorders and posttraumatic stress disorder. J. Clin. Psychiatry 61 (Suppl. 7), 22–32. 10795606

[ref13] Braun K. Bock J. (2011). The experience-dependent maturation of prefronto-limbic circuits and the origin of developmental psychopathology: implications for the pathogenesis and therapy of behavioural disorders. Dev. Med. Child Neurol. 53 (Suppl. 4), 14–18. 10.1111/j.1469-8749.2011.04056.x 21950388

[ref14] Brewerton T. D. (2007). Eating disorders, trauma, and comorbidity: focus on PTSD. Eat. Disord. 15, 285–304. 10.1080/10640260701454311, PMID: 17710567

[ref15] Briere J. Scott C. Weathers F. (2005). Peritraumatic and persistent dissociation in the presumed etiology of PTSD. Am. J. Psychiatry 162, 2295–2301. 10.1176/appi.ajp.162.12.2295, PMID: 16330593

[ref16] Briere J. Spinazzola J. (2005). Phenomenology and psychological assesment of complex posttraumatic states. J. Trauma. Stress. 18, 401–412. 10.1002/jts.20048 16281238

[ref17] Bromberg P. (1998a). Standing in spaces: Essays on dissociation, trauma and clinical process. (Hillsdale, NJ: The Analytic Press).

[ref18] Bromberg P. M. (1998b). Essay on clinical process, trauma and dissociation. (Hillsdale: The Analytic Press, Inc).

[ref19] Bromberg P. M. (2008). ““Mentalize this!” Dissociation, enactment and clinical process” in Mind to mind. Infant research, neuroscience and psychoanalysis. eds. Jurist E. L. Slade A. Bergner S. (New York: Other Press).

[ref20] Brown R. J. (2006). Different types of “dissociation” have different psychological mechanisms. J. Trauma Dissociation 7, 7–28. 10.1300/J229v07n04_02, PMID: 17182491

[ref21] Brown D. P. Scheflin A. W. Hammond D. C. (1998). Memory, trauma treatment, and the law. (New York: Norton).

[ref22] Bryant R. A. (2010). The complexity of complex PTSD. Am. J. Psychiatry 167, 879–881. 10.1176/appi.ajp.2010.10040606, PMID: 20693462

[ref23] Cakir S. Tasdelen Durak R. Ozyildirim I. Ince E. Sar V. (2016). Childhood trauma and treatment outcome in bipolar disorder. J. Trauma Dissociation 17, 397–409. 10.1080/15299732.2015.1132489, PMID: 26683845

[ref24] Cantor C. (2005). Evolution and posttraumatic stress: Disorders of vigilance and defence. (London/New York: Routledge).

[ref25] Cardeña E. Carlson E. (2011). Acute stress disorder revisited. Annu. Rev. Clin. Psychol. 7, 245–267. 10.1146/annurev-clinpsy-032210-104502, PMID: 21275643

[ref26] Carlier I. V. Hovens J. G. Streevelaar M. F. Van Rood Y. R. Van Veen T. (2016). Characteristics of suicidal outpatients with mood, anxiety and somatoform disorders: the role of childhood abuse and neglect. Int. J. Soc. Psychiatry 62, 316–326. 10.1177/0020764016629701 26896029

[ref27] Carlson E. A. Yates T. M. Sroufe L. A. (2009). “Dissociation and the development of the self” in Dissociation and dissociative disorders: DSM-V and beyond. eds. Dell P. O’Neil J. A. (New York: Routledge), 39–52.

[ref28] Carr C. P. Martins C. M. Stingel A. M. Lemgruber V. B. Juruena M. F. (2013). The role of early life stress in adult psychiatric disorders: a systematic review according to childhood trauma subtypes. J. Nerv. Ment. Dis. 201, 1007–1020. 10.1097/NMD.0000000000000049, PMID: 24284634

[ref29] Carter J. C. Bewell C. Blackmore E. Woodside D. B. (2006). The impact of childhood sexual abuse in anorexia nervosa. Child Abuse Negl. 30, 257–269. 10.1016/j.chiabu.2005.09.004, PMID: 16524628

[ref30] Caslini M. Bartoli F. Crocamo C. Dakanalis A. Clerici M. Carra G. (2016). Disentangling the association between child abuse and eating disorders: a systematic review and meta-analysis. Psychosom. Med. 78, 79–90. 10.1097/PSY.0000000000000233, PMID: 26461853

[ref31] Cassidy J. Shaver P. R. (2008). Handbook of attachment: Theory, research and clinical applications. (New York: Guilford Publications).

[ref32] Cattane N. Rossi R. Lanfredi M. Cattaneo A. (2017). Borderline personality disorder and childhood trauma: exploring the affected biological systems and mechanisms. BMC Psychiatry 17:221. 10.1186/s12888-017-1383-2 28619017 PMC5472954

[ref33] Choi K. R. Seng J. S. Briggs E. C. Munro-Kramer M. L. Graham-Bermann S. A. Lee R. C. (2017). The dissociative subtype of posttraumatic stress disorder (PTSD) among adolescents: co-occurring PTSD, depersonalization/derealization, and other dissociation symptoms. J. Am. Acad. Child Adolesc. Psychiatry 56, 1062–1072. 10.1016/j.jaac.2017.09.425 29173740 PMC5726572

[ref34] Chou C. Y. La Marca R. Steptoe A. Brewin C. R. (2018). Cardiovascular and psychological responses to voluntary recall of trauma in posttraumatic stress disorder. Eur. J. Psychotraumatol. 9:1472988. 10.1080/20008198.2018.1472988, PMID: 29887977 PMC5990938

[ref35] Chu J. A. (1998). Rebuilding shattered lives. the responsible treatment of complex post traumatic and dissociative disorders. (New York: Wiley & Sons).

[ref36] Chu J. A. (2010). Posttraumatic stress disorder: beyond DSM-IV. Am. J. Psychiatry 167, 615–617. 10.1176/appi.ajp.2010.10030310, PMID: 20516157

[ref37] Classen C. C. Pain C. Field N. P. Woods P. (2006). Posttraumatic personality disorder: a reformulation of complex posttraumatic stress disorder and borderline personality disorder. Psychiatr. Clin. North Am. 29, 87–112. viii-ix. 10.1016/j.psc.2005.11.001, PMID: 16530588

[ref38] Cloitre M. Courtois C. A. Charuvastra A. Carapezza R. Stolbach B. C. Green B. L. (2011). Treatment of complex PTSD: results of the ISTSS expert clinician survey on best practices. J. Trauma. Stress 24, 615–627. 10.1002/jts.20697, PMID: 22147449

[ref39] Cloitre M. Garvert D. W. Weiss B. Carlson E. B. Bryant R. A. (2014). Distinguishing PTSD, complex PTSD, and borderline personality disorder: a latent class analysis. Eur. J. Psychotraumatol. 5:25097. 10.3402/ejpt.v5.25097, PMID: 25279111 PMC4165723

[ref40] Cloitre M. Stolbach B. C. Herman J. L. Van der Kolk B. Pynoos R. Wang J.. (2009). A developmental approach to complex PTSD: childhood and adult cumulative trauma as predictors of symptom complexity. J. Trauma. Stress 22, 399–408. 10.1002/jts.20444, PMID: 19795402

[ref41] Cloitre M. Stovall-McClough K. C. Nooner K. Zorbas P. Cherry S. Jackson C. L.. (2010). Treatment for PTSD related to childhood abuse: a randomized controlled trial. Am. J. Psychiatry 167, 915–924. 10.1176/appi.ajp.2010.09081247, PMID: 20595411

[ref42] Courtois C. A. Ford J. D. (2009). Treating complex traumatic stress disorders. (New York/London: The Guilford Press).

[ref43] Courtois C. A. Ford J. D. Cloitre M. (2009). “Best practices in psychotherapy for adults” in Treating complex traumatic stress disorders. eds. Courtois C. A. Ford J. D. (New York/London: The Guilford Press), 82–103.

[ref44] Dalenberg C. Palesh O. (2010). “Scientific progress and methodological issues in the study of recovered and false memories of trauma” in The impact of early life trauma on health and disease: The hidden epidemic. eds. Lanius R. A. Vermetten E. Pain C. (New York: Cambridge University Press).

[ref45] de Aquino Ferreira L. F. Queiroz Pereira F. H. Neri Benevides A. M. L. Aguiar Melo M. C. (2018). Borderline personality disorder and sexual abuse: a systematic review. Psychiatry Res. 262, 70–77. 10.1016/j.psychres.2018.01.043, PMID: 29407572

[ref46] De Bellis M. D. Zisk A. (2014). The biological effects of childhood trauma. Child Adolesc. Psychiatr. Clin. N. Am. 23, 185–222. vii. 10.1016/j.chc.2014.01.002, PMID: 24656576 PMC3968319

[ref47] Dell P. F. O’Neil J. A. E. (2009). Dissociation and the dissociative disorders: DSM-V and beyond. (New York, NY, US: Routledge/Taylor & Francis Group).

[ref48] Denton R. Frogley C. Jackson S. John M. Querstret D. (2017). The assessment of developmental trauma in children and adolescents: a systematic review. Clin. Child Psychol. Psychiatry 22, 260–287. 10.1177/1359104516631607, PMID: 26940119

[ref49] Dimaggio G. Carcione A. Salvatore G. Semerari A. Nicolo G. (2010). A rational model for maximizing the effects of therapeutic relationship regulation in personality disorders with poor metacognition and over-regulation of affects. Psychol. Psychother. 83, 363–384. 10.1348/147608310X485256 25268484

[ref50] Dorahy M. J. Corry M. Shannon M. Macsherry A. Hamilton G. McRobert G.. (2009). Complex PTSD, interpersonal trauma and relational consequences: findings from a treatment-receiving Northern Irish sample. J. Affect. Disord. 112, 71–80. 10.1016/j.jad.2008.04.003, PMID: 18511130

[ref51] Dorahy M. J. van der Hart O. (2015). DSM-5’s posttraumatic stress disorder with dissociative symptoms: challenges and future directions. J. Trauma Dissociation 16, 7–28. 10.1080/15299732.2014.908806, PMID: 24983300

[ref52] Dorrington S. Zavos H. Ball H. McGuffin P. Rijsdijk F. Siribaddana S.. (2014). Trauma, post-traumatic stress disorder and psychiatric disorders in a middle-income setting: prevalence and comorbidity. Br. J. Psychiatry 205, 383–389. 10.1192/bjp.bp.113.141796, PMID: 25257062 PMC4217028

[ref53] Dutra L. Bureau J. F. Holmes B. Lyubchik A. Lyons-Ruth K. (2009). Quality of early care and childhood trauma: a prospective study of developmental pathways to dissociation. J. Nerv. Ment. Dis. 197, 383–390. 10.1097/NMD.0b013e3181a653b7, PMID: 19525736 PMC2697443

[ref54] Dworkin M. (2005). EMDR and the relational imperative: The therapeutic relationship in EMDR treatment. (New York: Routledge).

[ref55] Edalati H. Krank M. D. (2016). Childhood maltreatment and development of substance use disorders: a review and a model of cognitive pathways. Trauma Violence Abuse 17, 454–467. 10.1177/1524838015584370, PMID: 25964275

[ref56] Ellenberger H. F. (1970). The discovery of the unconscious. (New York: Basic Books).

[ref57] Fairbank J. A. Fairbank D. W. (2009). Epidemiology of child traumatic stress. Curr. Psychiatry Rep. 11, 289–295. 10.1007/s11920-009-0042-9, PMID: 19635237

[ref58] Fang X. Brown D. S. Florence C. S. Mercy J. A. (2012). The economic burden of child maltreatment in the United States and implications for prevention. Child Abuse Negl. 36, 156–165. 10.1016/j.chiabu.2011.10.006, PMID: 22300910 PMC3776454

[ref59] Farina B. Imperatori C. (2017). What if dissociation were a psychopathological dimension related to trauma? Authors’ reply to diagnostic challenges leading to underdiagnosis of dissociative disorders. Neuropsychiatr. Dis. Treat. 13, 409–410. 10.2147/NDT.S131439 PMC5308586 28223813

[ref60] Farina B. Liotti G. (2013). Does a dissociative psychopathological dimension exist? A review on dissociative processes and symptoms in developmental trauma spectrum disorders. Clin. Neuropsychiatry 10, 11–18.

[ref61] Farina B. Mazzotti E. Pasquini P. Nijenhuis E. Di Giannantonio M. (2011). Somatoform and psychoform dissociation among students. J. Clin. Psychol. 67, 665–672. 10.1002/jclp.20787, PMID: 21433009

[ref62] Farina B. Speranza A. M. Dittoni S. Gnoni V. Trentini C. Vergano C. M.. (2014). Memories of attachment hamper EEG cortical connectivity in dissociative patients. Eur. Arch. Psychiatry Clin. Neurosci. 264, 449–458. 10.1007/s00406-013-0461-9, PMID: 24121863

[ref63] Farina B. Speranza A. M. Imperatori C. Quintiliani M. I. Della Marca G. (2015). Change in heart rate variability after the adult attachment interview in dissociative patients. J. Trauma Dissociation 16, 170–180. 10.1080/15299732.2014.975309, PMID: 25492425

[ref64] Fegert J. M. Stotzel M. (2016). Child protection: a universal concern and a permanent challenge in the field of child and adolescent mental health. Child Adolesc. Psychiatry Ment. Health 10:18. 10.1186/s13034-016-0106-7 27303443 PMC4906729

[ref65] Fisher J. (2017). Healing the fragmented selves of trauma survivors: Overcoming internal self-alienation. (New York: Routledge).

[ref500] Fisher J. Ogden P. (2009). “Sensorimotor Psychotherapy” in Treating Complex Traumatic Stress Disorders. eds. Courtois C. A. Ford J. D. (New York, London: The Guilford Press), 312–318.

[ref66] Fonagy P. Target M. (2008). “Attachment, trauma, and psychoanalysis” in Mind to mind. eds. Jurist E. L. Slade A. Bergner S. (New York: Other Press), 15–42.

[ref67] Fontenelle L. F. Domingues A. M. Souza W. F. Mendlowicz M. V. de Menezes G. B. Figueira I. L.. (2007). History of trauma and dissociative symptoms among patients with obsessive-compulsive disorder and social anxiety disorder. Psychiatry Q. 78, 241–250. 10.1007/s11126-007-9043-1, PMID: 17453345

[ref68] Ford J. D. (2015). Complex PTSD: research directions for nosology/assessment, treatment, and public health. Eur. J. Psychotraumatol. 6:27584. 10.3402/ejpt.v6.27584, PMID: 25994023 PMC4439420

[ref69] Ford J. D. Courtois C. A. (2009). “Defining and understanding complex trauma and complex traumatic stress disorders” in Treating complex traumatic stress disorders. eds. Courtois C. A. Ford J. D. (New York/London: The Guilford Press), 13–30.

[ref70] Ford J. D. Courtois C. A. (2014). Complex PTSD, affect dysregulation, and borderline personality disorder. Borderline Personal Disord. Emot. Dysregul. 1:9. 10.1186/2051-6673-1-9, PMID: 26401293 PMC4579513

[ref71] Fosha D. (2003). “Dyadic regulation & experiential work with emotion & relatedness in trauma & disorganized attachment” in Healing trauma: Attachment, trauma, the brain, and the mind. eds. Solomon M. F. Siegel D. J. (New York: Norton), 221–281.

[ref72] Fosha D. Paivio S. C. Gleiser K. Ford J. D. (2009). “Experiential and emotion-focused therapy” in Treating complex traumatic stress disorders. eds. Courtois C. A. Ford J. D. (New York/London: The Guilford Press), 286–311.

[ref73] Frewen P. Lanius R. A. (2015). Healing the traumatized self: Consciousness, neuroscience, and treatment. (New York: W. W. Norton & Co).

[ref74] Gander M. Buchheim A. (2015). Attachment classification, psychophysiology and frontal EEG asymmetry across the lifespan: a review. Front. Hum. Neurosci. 9:79. 10.3389/fnhum.2015.00079, PMID: 25745393 PMC4333768

[ref75] Gilbert R. Widom C. S. Browne K. Fergusson D. Webb E. Janson S. (2009). Burden and consequences of child maltreatment in high-income countries. Lancet 373, 68–81. 10.1016/S0140-6736(08)61706-7, PMID: 19056114

[ref76] Gleiser K. Ford J. D. Fosha D. (2008). Contrasting exposure and experiential therapies for complex posttraumatic stress disorder. Psychotherapy 45, 340–360. 10.1037/a0013323, PMID: 22122495

[ref77] Granqvist P. Sroufe L. A. Dozier M. Hesse E. Steele M. van Ijzendoorn M.. (2017). Disorganized attachment in infancy: a review of the phenomenon and its implications for clinicians and policy-makers. Attach Hum. Dev. 19, 534–558. 10.1080/14616734.2017.1354040, PMID: 28745146 PMC5600694

[ref78] Green J. G. McLaughlin K. A. Berglund P. A. Gruber M. J. Sampson N. A. Zaslavsky A. M.. (2010). Childhood adversities and adult psychiatric disorders in the national comorbidity survey replication I: associations with first onset of DSM-IV disorders. Arch. Gen. Psychiatry 67, 113–123. 10.1001/archgenpsychiatry.2009.186, PMID: 20124111 PMC2822662

[ref79] Habetha S. Bleich S. Weidenhammer J. Fegert J. M. (2012). A prevalence-based approach to societal costs occurring in consequence of child abuse and neglect. Child Adolesc. Psychiatry Ment. Health 6:35. 10.1186/1753-2000-6-35, PMID: 23158382 PMC3540003

[ref80] Harkness K. L. Bagby R. M. Kennedy S. H. (2012). Childhood maltreatment and differential treatment response and recurrence in adult major depressive disorder. J. Consult. Clin. Psychol. 80, 342–353. 10.1037/a0027665, PMID: 22428942

[ref81] Harlow H. F. Zimmerman R. (1959). Affectional responses in the infant monkey. Science 130, 421–432. 10.1126/science.130.3373.421, PMID: 13675765

[ref82] Herman J. L. (1992a). Complex PTSD: a syndrome in survivors of prolonged and repeated trauma. J. Trauma. Stress. 5, 377–391. 10.1002/jts.2490050305

[ref83] Herman J. L. (1992b). Trauma and recovery. (New York: Basic Books).

[ref84] Herman J. L. (2009). “Foreword” in Treating complex traumatic stress disorders. eds. Courtois C. Ford J. D. (New York: Guilfrod Press), xiii–xvii.

[ref85] Holmes E. A. Brown R. J. Mansell W. Fearon R. P. Hunter E. C. Frasquilho F.. (2005). Are there two qualitatively distinct forms of dissociation? A review and some clinical implications. Clin. Psychol. Rev. 25, 1–23. 10.1016/j.cpr.2004.08.006, PMID: 15596078

[ref86] Hopper A. Ciorciari J. Johnson G. Spensley J. Sergejew A. Stough C. (2002). EEG coherence and dissociative identity disorder: comparing EEG coherence in DID hosts, alters, controls and acted alters. J. Trauma Dissociation 3, 75–88. 10.1300/J229v03n01_06

[ref87] Howell E. F. (2005). The dissociative mind. (Hillsdale: The Analytic Press).

[ref88] Howell E. F. (2011). Understanding and treating dissociative identity disorder: A relational approach. (New York: Routledge).

[ref89] Isobel S. Goodyear M. Foster K. (2017). Psychological trauma in the context of familial relationships: a concept analysis. Trauma Violence Abuse, 1524838017726424. 10.1177/1524838017726424 29333976

[ref90] Janet P. (1889). L’automatisme Psychologique: Essai de Psychologie Expérimentale sur les Formes Inférieures de l’Activité Humaine. (Paris: Alcan).

[ref91] Jaworska-Andryszewska P. Rybakowski J. K. (2018). Childhood trauma in mood disorders: neurobiological mechanisms and implications for treatment. Pharmacol. Rep. 71, 112–120. 10.1016/j.pharep.2018.10.004 30544098

[ref92] Jepsen E. K. Langeland W. Heir T. (2014). Early traumatized inpatients high in psychoform and somatoform dissociation: characteristics and treatment response. J. Trauma Dissociation 15, 572–587. 10.1080/15299732.2014.924461, PMID: 24983399

[ref93] Jobst A. Brakemeier E. L. Buchheim A. Caspar F. Cuijpers P. Ebmeier K. P.. (2016). European Psychiatric Association Guidance on psychotherapy in chronic depression across Europe. Eur. Psychiatry 33, 18–36. 10.1016/j.eurpsy.2015.12.003, PMID: 26854984

[ref94] Johnstone J. M. Luty S. E. Carter J. D. Mulder R. T. Frampton C. M. Joyce P. R. (2009). Childhood neglect and abuse as predictors of antidepressant response in adult depression. Depress. Anxiety 26, 711–717. 10.1002/da.20590, PMID: 19544315

[ref95] Kajeepeta S. Gelaye B. Jackson C. L. Williams M. A. (2015). Adverse childhood experiences are associated with adult sleep disorders: a systematic review. Sleep Med. 16, 320–330. 10.1016/j.sleep.2014.12.013 25777485 PMC4635027

[ref96] Kessler R. C. Sonnega A. Bromet E. (1995). Posttraumatic stress disorder in the national comorbidity survey. Arch. Gen. Psychiatry 52, 1048–1060. 10.1001/archpsyc.1995.03950240066012, PMID: 7492257

[ref97] Kinsler P. J. Courtois C. A. Frankel A. S. (2009). “Therapeutic alliance and risk management” in Treating complex traumatic stress disorders. eds. Courtois C. A. Ford J. D. (New York/London: The Guilford Press), 183–201.

[ref98] Konkoly Thege B. Horwood L. Slater L. Tan M. C. Hodgins D. C. Wild T. C. (2017). Relationship between interpersonal trauma exposure and addictive behaviors: a systematic review. BMC Psychiatry 17:164. 10.1186/s12888-017-1323-1 28472931 PMC5418764

[ref99] Korn D. L. Leeds A. M. (2002). Preliminary evidence of efficacy for EMDR resource development and installation in the stabilization phase of treatment of complex posttraumatic stress disorder. J. Clin. Psychol. 58, 1465–1487. 10.1002/jclp.10099 12455016

[ref100] Krystal H. (1988). Integration and self-healing affect-trauma-alexithymia. (Hillsdale: The Analync Press).

[ref101] La Mela C. Maglietta M. Castellini G. Amoroso L. Lucarelli S. (2010). Dissociation in eating disorders: relationship between dissociative experiences and binge-eating episodes. Compr. Psychiatry 51, 393–400. 10.1016/j.comppsych.2009.09.008, PMID: 20579513

[ref102] Lanius R. A. Boyd J. E. McKinnon M. C. Nicholson A. A. Frewen P. Vermetten E. (2018). A Review of the neurobiological basis of trauma-related dissociation and its relation to cannabinoid- and opioid-mediated stress response: a transdiagnostic, translational approach. Curr. Psychiatry Rep. 20:118. 10.1007/s11920-018-0983-y 30402683

[ref103] Lanius R. A. Vermetten E. Loewenstein R. J. Brand B. Schmahl C. Bremner J. D. (2010a). Emotion modulation in PTSD: clinical and neurobiological evidence for a dissociative subtype. Am. J. Psychiatry 167, 640–647. 10.1176/appi.ajp.2009.09081168 20360318 PMC3226703

[ref104] Lanius R. A. Vermetten E. Pain C. (2010b). The impact of early relational trauma on helath and disease. The hidden epidemic. (Cambridge: Cambridge University Press).

[ref105] Lanius R. A. Williamson P. C. Bluhm R. L. Densmore M. Boksman K. Neufeld R. W.. (2005). Functional connectivity of dissociative responses in posttraumatic stress disorder: a functional magnetic resonance imaging investigation. Biol. Psychiatry 57, 873–884. 10.1016/j.biopsych.2005.01.011, PMID: 15820708

[ref106] Lewis K. L. Grenyer B. F. (2009). Borderline personality or complex posttraumatic stress disorder? an update on the controversy. Harv. Rev. Psychiatry 17, 322–328. 10.3109/10673220903271848, PMID: 19832046

[ref107] Lewis C. C. Simons A. D. Nguyen L. J. Murakami J. L. Reid M. W. Silva S. G.. (2010). Impact of childhood trauma on treatment outcome in the Treatment for Adolescents with Depression Study (TADS). J. Am. Acad. Child Adolesc. Psychiatry 49, 132–140. 10.1016/j.jaac.2009.10.007, PMID: 20215935

[ref108] Liotti G. (1992). Disorganized attachment in the etiology of the dissociative disorders. Dissociation 5, 196–204.

[ref109] Liotti G. (2004). Trauma, dissociation and disorganized attachment: three strands of a single braid. Psychother. Theory Res. Pract. Train. 41, 472–486. 10.1037/0033-3204.41.4.472

[ref110] Liotti G. (2009). “Attachment and dissociation” in Dissociation and dissociative disorders: DSM-V and beyond. eds. Dell P. O’Neil J. A. (New York: Routledge), 53–65.

[ref111] Liotti G. (2017). Conflicts between motivational systems related to attachment trauma: key to understanding the intra-family relationship between abused children and their abusers. J. Trauma Dissociation 18, 304–318. 10.1080/15299732.2017.1295392, PMID: 28318416

[ref112] Liotti G. Cortina M. Farina B. (2008). Attachment theory and multiple integrated treatments of borderline patients. J. Am. Acad. Psychoanal. Dyn. Psychiatry 36, 295–315. 10.1521/jaap.2008.36.2.295 18593257

[ref113] Liotti G. Farina B. (2016). “Painful incoherence: the self in borderline personality disorder” in The self in understanding and treating psychological disorders. eds. Kyrios M. Moulding R. Nedeljkovic M. Bhar S. S. Doron G. Mikulincer M. (Cambridge: Cambridge University Press), 169–178.

[ref114] Liotti G. Gilbert P. (2011). Mentalizing, motivation and social mentalities: theoretical considerations and implications for psychotherapy. Psychol. Psychother. 84, 9–25. 10.1348/147608310X520094 22903828

[ref115] Liotti G. Liotti M. (2019). “Reflections on some contributions to contemporary psychotraumatology in the light of Janet’s critique of Freud’s theories” in Rediscovering Pierre Janet: His relevance for psychoanalysis, psychotraumatology, and psychotherapy. eds. Craparo G. Ortu F. van der Hart O. (London: Routledge).

[ref116] Liotti G. Prunetti E. (2010). “Metacognitive deficits in traumarelated disorders: contingent on interpersonal motivational contexts?” in Metacognition and severe adult mental disorders: From basic research to treatment. (London: Routledge), 196–214.

[ref117] Loftus E. (2003). Our changeable memories: legal and practical implications. Nat. Rev. Neurosci. 4, 231–234. 10.1038/nrn1054, PMID: 12612635

[ref118] Lorenz K. (1949). King Solomon’s Ring. En transl (1952). (New York: Crowell).

[ref119] Main M. Hesse E. (1990). “Parents’ unresolved traumatic experiences are related to infant disorganized attachment status: is frightened/frightening parental behavior the linking mechanism?” in Attachment in the preschool years. eds. Greenberg M. Cichetti D. Cummings M. (Chicago: University of Chicago Press), 121–160.

[ref120] Main M. Solomon J. (1986). “Discovery of a new, insecure-disorganized/disoriented attachment pattern” in Affective development in infancy. eds. Brazelton T. B. Yogman M. (Norwood, New Jersey: Ablex), 95–124.

[ref121] Maj M. (2005). “Psychiatric comorbidity”: an artefact of current diagnostic systems? Br. J. Psychiatry 186, 182–184. 10.1192/bjp.186.3.182 15738496

[ref122] Mandelli L. Petrelli C. Serretti A. (2015). The role of specific early trauma in adult depression: a meta-analysis of published literature. Childhood trauma and adult depression. Eur. Psychiatry 30, 665–680. 10.1016/j.eurpsy.2015.04.007, PMID: 26078093

[ref123] Mayo D. Corey S. Kelly L. H. Yohannes S. Youngquist A. L. Stuart B. K. (2017). The role of trauma and stressful life events among individuals at clinical high risk for psychosis: a review. Front. Psychiatry 8:55. 10.3389/fpsyt.2017.00055 28473776 PMC5397482

[ref124] Mazzotti E. Farina B. Imperatori C. Mansutti F. Prunetti E. Speranza A. M.. (2016). Is the Dissociative Experiences Scale able to identify detachment and compartmentalization symptoms? Factor structure of the Dissociative Experiences Scale in a large sample of psychiatric and nonpsychiatric subjects. Neuropsychiatr. Dis. Treat. 12, 1295–1302. 10.2147/NDT.S105110, PMID: 27350746 PMC4902245

[ref125] McBride C. Atkinson L. Quilty L. C. Bagby R. M. (2006). Attachment as moderator of treatment outcome in major depression: a randomized control trial of interpersonal psychotherapy versus cognitive behavior therapy. J. Consult. Clin. Psychol. 74, 1041–1054. 10.1037/0022-006X.74.6.1041, PMID: 17154734

[ref126] McCrory E. J. Gerin M. I. Viding E. (2017). Annual research review: childhood maltreatment, latent vulnerability and the shift to preventative psychiatry – the contribution of functional brain imaging. J. Child Psychol. Psychiatry 58, 338–357. 10.1111/jcpp.12713, PMID: 28295339 PMC6849838

[ref127] Meares R. (1999). The contribution of Hughlings Jackson to an understanding of dissociation. Am. J. Psychiatry 156, 1850–1855. 10.1176/ajp.156.12.1850, PMID: 10588396

[ref128] Meares R. (2000). Intimacy and alienation. (London: Routledge).

[ref129] Meares R. (2012). The dissociation model of borderline personality disorder. (New York: Norton Professional Books).

[ref130] Meares R. Barral C. (2019). “The holistic project of Pierre Janet, part one: disintegration or désagrégation” in Rediscovering Pierre Janet: His relevance for psychoanalysis, psychotraumatology, and psychotherapy. eds. Craparo G. Ortu F. van der Hart O. (London: Routledge).

[ref131] Michelson L. June K. Vives A. Testa S. Marchione N. (1998). The role of trauma and dissociation in cognitive-behavioral psychotherapy outcome and maintenance for panic disorder with agoraphobia. Behav. Res. Ther. 36, 1011–1050. 10.1016/S0005-7967(98)00073-4, PMID: 9737056

[ref132] Misiak B. Frydecka D. (2016). A history of childhood trauma and response to treatment with antipsychotics in first-episode schizophrenia patients: preliminary results. J. Nerv. Ment. Dis. 204, 787–792. 10.1097/NMD.0000000000000567, PMID: 27441460

[ref133] Nardo D. Hogberg G. Looi J. C. Larsson S. Hallstrom T. Pagani M. (2010). Gray matter density in limbic and paralimbic cortices is associated with trauma load and EMDR outcome in PTSD patients. J. Psychiatr. Res. 44, 477–485. 10.1016/j.jpsychires.2009.10.014, PMID: 19942229

[ref134] Newman J. M. Turnbull A. Berman B. A. Rodrigues S. Serper M. R. (2010). Impact of traumatic and violent victimization experiences in individuals with schizophrenia and schizoaffective disorder. J. Nerv. Ment. Dis. 198, 708–714. 10.1097/NMD.0b013e3181f49bf1, PMID: 20921860

[ref135] Nijenhuis E. R. (in press). The metaphor of dissociation: teleological, phenomenological, structural, dynamical. Quad. Psicol. Cog.

[ref136] Nijenhuis E. R. Spinhoven P. Vanderlinden J. Van Dyck R. Van der Hart O. (1998). Somatoform dissociative symptoms as related to animal defensive reactions to predatory imminence and injury. J. Abnorm. Psychol. 107, 63–73. 10.1037/0021-843X.107.1.63, PMID: 9505039

[ref137] Nijenhuis E. R. Van der Hart O. (2011). Dissociation in trauma: a new definition and comparison with previous formulations. J. Trauma Dissociation 12, 416–445. 10.1080/15299732.2011.570592, PMID: 21667387

[ref138] Nijenhuis E. R. Van Dyck R. Kuile M. M. Mourits M. J. Spinhoven P. Van der Hart O. (2003). Evidence for associations among somatoform dissociation, psychological dissociation and reported trauma in patients with chronic pelvic pain. J. Psychosom. Obstet. Gynaecol. 24, 87–98. 10.3109/01674820309042806 12854393

[ref139] Ogawa J. R. Sroufe L. A. Weinfield N. S. Carlson E. A. Egeland B. (1997). Development and the fragmented self: longitudinal study of dissociative symptomatology in a non-clinical samples. Dev. Psychopathol. 9, 855–879. 10.1017/S0954579497001478, PMID: 9449009

[ref140] Ogden P. Fisher J. (2015). Sensorimotor psychotherapy: Interventions for trauma and attachment. (New York: W. W. Norton & Co).

[ref141] Ogden P. Pain C. Fisher J. (2006). A sensorimotor approach to the treatment of trauma and dissociation. Psychiatr. Clin. North Am. 29, 263–279, xi-xii. 10.1016/j.psc.2005.10.012, PMID: 16530597

[ref142] Opler L. A. Grennan M. S. Ford J. D. (2009). “Pharmacotherapy” in Treating complex traumatic stress disorders. eds. Courtois C. A. Ford J. D. (New York/London: The Guilford Press), 329–349.

[ref143] Pagura J. Stein M. B. Bolton J. M. Cox B. J. Grant B. Sareen J. (2010). Comorbidity of borderline personality disorder and posttraumatic stress disorder in the U.S. population. J. Psychiatr. Res. 44, 1190–1198. 10.1016/j.jpsychires.2010.04.016, PMID: 20537660 PMC4209725

[ref144] Pelcovitz D. Van der Kolk B. Roth S. Mandel F. Kaplan S. Resick P. (1997). Development of a criteria set and a structured interview for disorders of extreme stress (SIDES). J. Trauma. Stress 10, 3–16. 10.1002/jts.2490100103, PMID: 9018674

[ref145] Pessoa L. (2017). A network model of the emotional brain. Trends Cogn. Sci. 21, 357–371. 10.1016/j.tics.2017.03.002, PMID: 28363681 PMC5534266

[ref146] Picardi A. Pallagrosi M. Fonzi L. Biondi M. (2017). Psychopathological dimensions and the clinician’s subjective experience. Psychiatry Res. 258, 407–414. 10.1016/j.psychres.2017.08.079, PMID: 28870646

[ref147] Pignatelli A. M. Wampers M. Loriedo C. Biondi M. Vanderlinden J. (2017). Childhood neglect in eating disorders: a systematic review and meta-analysis. J. Trauma Dissociation 18, 100–115. 10.1080/15299732.2016.1198951 27282982

[ref148] Porges S. W. (2007). The polyvagal perspective. Biol. Psychol. 74, 116–143. 10.1016/j.biopsycho.2006.06.009, PMID: 17049418 PMC1868418

[ref149] Prasko J. Grambal A. Kasalova P. Kamardova D. Ociskova M. Holubova M. (2016). Impact of dissociation on treatment of depressive and anxiety spectrum disorders with and without personality disorders. Neuropsychiatr. Dis. Treat. 12, 2659–2676. 10.2147/NDT.S118058 27799774 PMC5074730

[ref150] Propper R. E. Pierce J. Geisler M. W. Christman S. D. Bellorado N. (2007). Effect of bilateral eye movements on frontal interhemispheric gamma EEG coherence: implications for EMDR therapy. J. Nerv. Ment. Dis. 195, 785–788. 10.1097/NMD.0b013e318142cf73, PMID: 17984782

[ref151] Read J. van Os J. Morrison A. P. Ross C. A. (2005). Childhood trauma, psychosis and schizophrenia: a literature review with theoretical and clinical implications. Acta Psychiatr. Scand. 112, 330–350. 10.1111/j.1600-0447.2005.00634.x, PMID: 16223421

[ref152] Resick P. A. Nishith P. Griffin M. G. (2003). How well does cognitive-behavioral therapy treat symptoms of complex PTSD? An examination of child sexual abuse survivors within a clinical trial. CNS Spectr. 8, 340–355. 10.1017/S1092852900018605 12766690 PMC2970926

[ref153] Roelofs K. Pasman J. (2016). Stress, childhood trauma, and cognitive functions in functional neurologic disorders. Handb. Clin. Neurol. 139, 139–155. 10.1016/B978-0-12-801772-2.00013-8 27719835

[ref154] Ross C. A. (2009). “The theory of a dissociative subtype of schizophrenia” in Dissociation and dissociative disorders: DSM-V and beyond. eds. Dell P. O’Neil J. A. (New York: Routledge), 557–568.

[ref155] Roth S. Newman E. Pelcovitz D. van der Kolk B. Mandel F. S. (1997). Complex PTSD in victims exposed to sexual and physical abuse: results from the DSM-IV field trial for posttraumatic stress disorder. J. Trauma. Stress 10, 539–555. 10.1002/jts.2490100403, PMID: 9391940

[ref156] Rufer M. Held D. Cremer J. Fricke S. Moritz S. Peter H. (2006). Dissociation as a predictor of cognitive behavior therapy outcome in patients with obsessive-compulsive disorder. Psychother. Psychosom. 75, 40–46. 10.1159/000089225 16361873

[ref157] Safran J. D. Muran J. C. (2000). Negotiating the therapeutic alliance: A relational treatment guide. (New York: Guilford Press).

[ref158] Sar V. (2011). Developmental trauma, complex PTSD, and the current proposal of DSM-5. Eur. J. Psychotraumatol. 2:1. 10.3402/ejpt.v2i0.5622, PMID: 22893823 PMC3402152

[ref159] Sar V. (2017). Parallel-distinct structures of internal world and external reality: disavowing and re-claiming the self-identity in the aftermath of trauma-generated dissociation. Front. Psychol. 8:216. 10.3389/fpsyg.2017.00216, PMID: 28261144 PMC5313499

[ref160] Sar V. Akyuz G. Kundakci T. Kiziltan E. Dogan O. (2004). Childhood trauma, dissociation, and psychiatric comorbidity in patients with conversion disorder. Am. J. Psychiatry 161, 2271–2276. 10.1176/ajp.161.12.2271, PMID: 15569899

[ref161] Sar V. Dorahy M. J. Kruger C. (2017). Revisiting the etiological aspects of dissociative identity disorder: a biopsychosocial perspective. Psychol. Res. Behav. Manag. 10, 137–146. 10.2147/PRBM.S113743 28496375 PMC5422461

[ref162] Schilling C. Weidner K. Schellong J. Joraschky P. Pohlmann K. (2015). Patterns of childhood abuse and neglect as predictors of treatment outcome in inpatient psychotherapy: a typological approach. Psychopathology 48, 91–100. 10.1159/000368121, PMID: 25501445

[ref163] Schimmenti A. (2017). The developmental roots of dissociation: a multiple mediation analysis. Psychoanal. Psychol. 34, 96–105. 10.1037/pap0000084

[ref164] Schimmenti A. Sar V. (2019). A correlation network analysis of dissociative experiences. J. Trauma Dissociation 1–18. 10.1080/15299732.2019.1572045, PMID: 30714885

[ref165] Schore A. N. (2009). “Attachment trauma and the developing of right brain: origin of pathological dissociation” in Dissociation and dissociative disorders: DSM-V and beyond. eds. Dell P. O’Neil J. A. (New York: Routledge).

[ref166] Schubert S. Lee C. (2009). Adult PTSD and its treatment with EMDR: a review of controversies, evidence and theoretical knowledge. J. EMDR Pract. Res. 3, 117–132. 10.1891/1933-3196.3.3.117

[ref167] Semiz U. B. Inanc L. Bezgin C. H. (2014). Are trauma and dissociation related to treatment resistance in patients with obsessive-compulsive disorder? Soc. Psychiatry Psychiatr. Epidemiol. 49, 1287–1296. 10.1007/s00127-013-0787-7, PMID: 24213522

[ref168] Shapiro F. (2002). EMDR 12 years after its introduction: past and future research. J. Clin. Psychol. 58, 1–22. 10.1002/jclp.1126, PMID: 11748594

[ref169] Solomon J. George C. C. (2011). Disorganized attachment and caregiving. (New York: Guilford Publications).

[ref170] Sommerfeld E. Orbach I. Zim S. Mikulincer M. (2008). An in-session exploration of ruptures in working alliance and their associations with clients’ core conflictual relationship themes, alliance-related discourse, and clients’ postsession evaluations. Psychother. Res. 18, 377–388. 10.1080/10503300701675873, PMID: 18815990

[ref171] Spermon D. Darlington Y. Gibney P. (2010). Psychodynamic psychotherapy for complex trauma: targets, focus, applications, and outcomes. Psychol. Res. Behav. Manag. 3, 119–127. 10.2147/PRBM.S10215, PMID: 22110335 PMC3218759

[ref172] Spinazzola J. Ford J. D. Zucker M. Van der Kolk B. A. Silva S. Smith S. F. (2005). Survey evaluates complex trauma exposure, outcome, and intervention among children and adolescents. Psychiatr. Ann. 35, 433–439. 10.3928/00485713-20050501-09

[ref173] Spitzer C. Barnow S. Freyberger H. J. Grabe H. J. (2007). Dissociation predicts symptom-related treatment outcome in short-term inpatient psychotherapy. Aust. N. Z. J. Psychiatry 41, 682–687. 10.1080/00048670701449146 17620165

[ref174] Spitzer C. Barnow S. Wingenfeld K. Rose M. Lowe B. Grabe H. J. (2009). Complex post-traumatic stress disorder in patients with somatization disorder. Aust. N. Z. J. Psychiatry 43, 80–86. 10.1080/00048670802534366, PMID: 19085532

[ref175] Steele K. van der Hart O. Nijenhuis E. R. S. (2001). Dependency in the treatment of complex posttraumatic stress disorder and dissociative disorders. J. Trauma Dissociation 2, 79–116. 10.1300/J229v02n04_05 29389299

[ref176] Steele K. van der Hart O. Nijenhuis E. R. (2005). Phase-oriented treatment of structural dissociation in complex traumatization: overcoming trauma-related phobias. J. Trauma Dissociation 6, 11–53. 10.1300/J229v06n03_02, PMID: 16172081

[ref177] Stein D. J. van der Kolk B. A. Austin C. Fayyad R. Clary C. (2006). Efficacy of sertraline in posttraumatic stress disorder secondary to interpersonal trauma or childhood abuse. Ann. Clin. Psychiatry 18, 243–249. 10.1080/10401230600948431, PMID: 17162624

[ref178] Stern D. (2008). “On having to find what you don’t know how to look for: two perspectives on reflection” in Mind to mind. Infant research, neuroscience and psychoanalysis. eds. Jurist E. L. Slade A. Bergner S. (New York: Other Press), 398–407.

[ref179] Stoltenborgh M. Bakermans-Kranenburg M. J. Alink L. R. Van IJzendoorn M. H. (2012). The universality of childhood emotional abuse: a meta-analysis of worldwide prevalence. J. Aggress. Maltreat. Trauma 21, 870–890. 10.1080/10926771.2012.708014

[ref180] Stoltenborgh M. Bakermans-Kranenburg M. J. Van IJzendoorn M. H. (2013a). The neglect of child neglect: a meta-analytic review of the prevalence of neglect. Soc. Psychiatry Psychiatr. Epidemiol. 48, 345–355. 10.1007/s00127-012-0549-y 22797133 PMC3568479

[ref181] Stoltenborgh M. Bakermans-Kranenburg M. J. Van IJzendoorn M. H. Alink L. R. (2013b). Cultural-geographical differences in the occurrence of child physical abuse? A meta-analysis of global prevalence. Int. J. Psychol. 48, 81–94. 10.1080/00207594.2012.697165 23597008

[ref182] Stoltenborgh M. Van IJzendoorn M. H. Euser E. M. Bakermans-Kranenburg M. J. (2011). A global perspective on child sexual abuse: meta-analysis of prevalence around the world. Child Maltreat. 16, 79–101. 10.1177/1077559511403920, PMID: 21511741

[ref183] Szajnberg N. Goldenberg A. Harari U. (2010). “Early trauma later outcome: results from longitudinal studies and clinical observations” in The impact of early relational trauma on health and disease. The hidden epidemic. eds. Lanius R. A. Vermetten E. Pain C.. 2009/05/12 ed (Cambridge: Cambridge University Press), 33–42.

[ref184] Teicher M. H. Rabi K. Sheu Y. S. Serafine S. B. Andersen S. L. Anderson C. M. (2010). “Neurobiology of childhood trauma and adversity” in The impact of early relational trauma on helath and disease. The hidden epidemic. eds. Lanius R. A. Vermetten E. Pain C. (Cambridge: Cambridge University Press), 112–122.

[ref185] Teicher M. H. Samson J. A. Anderson C. M. Ohashi K. (2016). The effects of childhood maltreatment on brain structure, function and connectivity. Nat. Rev. Neurosci. 17, 652–666. 10.1038/nrn.2016.111, PMID: 27640984

[ref186] Terock J. Van der Auwera S. Janowitz D. Spitzer C. Barnow S. Miertsch M. (2016). from childhood trauma to adult dissociation: the role of PTSD and alexithymia. J. Anxiety Disord. 49, 374–382. 10.1159/000449004 27623153

[ref187] Thase M. E. (2009). Atypical depression: useful concept, but it’s time to revise the DSM-IV criteria. Neuropsychopharmacology 34, 2633–2641. 10.1038/npp.2009.100, PMID: 19741592

[ref188] Trottier K. MacDonald D. E. (2017). Update on psychological trauma, other severe adverse experiences and eating disorders: state of the research and future research directions. Curr. Psychiatry Rep. 19:45. 10.1007/s11920-017-0806-6 28624866

[ref189] U.S. Department of Health & Human Services, A.f.C.a.F., Administration on Children, Youth and Families, Children’s Bureau. (2017). Child maltreatment 2015. Available at: http://www.acf.hhs.gov/programs/cb/research-data-technology/statistics-research/child-maltreatment (Accessed December 31, 2018).

[ref190] Van der Hart O. Dorahy M. (2006). Pierre Janet and the concept of dissociation. Am. J. Psychiatry 163:1646; author reply 1646. 10.1176/ajp.2006.163.9.1646a, PMID: 16946201

[ref191] Van der Hart O. Dorahy M. (2009). “History of the concept of dissociation” in Dissociation and dissociative disorders: DSM-V and beyond. eds. Dell P. O’Neil J. A. (New York: Routledge).

[ref192] Van der Hart O. Nijenhuis E. R. Steele K. (2005). Dissociation: an insufficiently recognized major feature of complex posttraumatic stress disorder. J. Trauma. Stress 18, 413–423. 10.1002/jts.20049, PMID: 16281239

[ref193] Van der Hart O. Nijenhuis E. Steele K. (2006). The haunted self: Structural dissociation and the treatment of chronic traumatization. (New York/London: Norton).

[ref194] Van der Kolk B. A. (1996). “The complexity of adaptation to trauma: self-regulation, stimulus discrimination and characterological development” in Traumatic stress: The effects of overwhelming experience on mind, body, and society. eds. van der Kolk B. A. McFarlane A. C. Weisaeth L. (New York: Guilford Press), 182–213.

[ref195] Van der Kolk B. A. (2005). Developmental trauma disorder: toward a rational diagnosis for children with complex trauma histories. Psychiatr. Ann. 35, 401–408. 10.3928/00485713-20050501-06

[ref196] Van der Kolk B. (2014). The body keeps the score: Mind, brain and body in the transformation of trauma. (London: Penguin).

[ref197] Van der Kolk B. A. Pelcovitz D. Roth S. Mandel F. S. McFarlane A. Herman J. L. (1996). Dissociation, somatization, and affect dysregulation: the complexity of adaptation of trauma. Am. J. Psychiatry 153 (Suppl. 7), 83–93. 10.1176/ajp.153.7.83 8659645

[ref198] Van der Kolk B. A. Roth S. Pelcovitz D. Sunday S. Spinazzola J. (2005). Disorders of extreme stress: the empirical foundation of a complex adaptation to trauma. J. Trauma. Stress 18, 389–399. 10.1002/jts.20047, PMID: 16281237

[ref199] Van der Kolk B. A. Spinazzola J. Blaustein M. E. Hopper J. W. Hopper E. K. Korn D. L.. (2007). A randomized clinical trial of eye movement desensitization and reprocessing (EMDR), fluoxetine, and pill placebo in the treatment of posttraumatic stress disorder: treatment effects and long-term maintenance. J. Clin. Psychiatry 68, 37–46. 10.4088/JCP.v68n0105, PMID: 17284128

[ref200] Van Huijstee J. Vermetten E. (2018). The dissociative subtype of post-traumatic stress disorder: research update on clinical and neurobiological features. Curr. Top. Behav. Neurosci. 38, 229–248. 10.1007/7854_2017_33, PMID: 29063485

[ref201] Vanderlinden J. Vandereycken W. Van Dyck R. Vertommen H. (1993). Dissociative experiences and trauma in eating disorders. Int. J. Eat. Disord. 13, 187–193. 10.1002/1098-108X(199303)13:2<187::AID-EAT2260130206>3.0.CO;2-9, PMID: 8477287

[ref202] Vogel M. Braungardt T. Grabe H. J. Schneider W. Klauer T. (2013). Detachment, compartmentalization, and schizophrenia: linking dissociation and psychosis by subtype. J. Trauma Dissociation 14, 273–287. 10.1080/15299732.2012.724760, PMID: 23627477

[ref203] Vogel M. Spitzer C. Barnow S. Freyberger H. J. Grabe H. J. (2006). The role of trauma and PTSD-related symptoms for dissociation and psychopathological distress in inpatients with schizophrenia. Psychopathology 39, 236–242. 10.1159/000093924, PMID: 16778454

[ref204] Vrticka P. Vuilleumier P. (2012). Neuroscience of human social interactions and adult attachment style. Front. Hum. Neurosci. 6:212. 10.3389/fnhum.2012.00212, PMID: 22822396 PMC3398354

[ref205] Waller G. (1997). Drop-out and failure to engage in individual outpatient cognitive behavior therapy for bulimic disorders. Int. J. Eat. Disord. 22, 35–41. 10.1002/(SICI)1098-108X(199707)22:1<35::AID-EAT4>3.0.CO;2-3 9140733

[ref206] Waller N. G. Putnam F. W. Carlson E. B. (1996). Types of dissociation and dissociative types: a taxometric analysis of dissociative experiences. Psychol. Methods 1, 300–321. 10.1037/1082-989X.1.3.300

[ref207] Williams J. Bucci S. Berry K. Varese F. (2018). Psychological mediators of the association between childhood adversities and psychosis: a systematic review. Clin. Psychol. Rev. 65, 175–196. 10.1016/j.cpr.2018.05.009 30243100

[ref208] Williamson V. Creswell C. Fearon P. Hiller R. M. Walker J. Halligan S. L. (2017). The role of parenting behaviors in childhood post-traumatic stress disorder: a meta-analytic review. Clin. Psychol. Rev. 53, 1–13. 10.1016/j.cpr.2017.01.005, PMID: 28137661

[ref209] Witt A. Brown R. C. Plener P. L. Brahler E. Fegert J. M. (2017). Child maltreatment in Germany: prevalence rates in the general population. Child Adolesc. Psychiatry Ment. Health 11:47. 10.1186/s13034-017-0185-0 28974983 PMC5621113

[ref210] Witt A. Glaesmer H. Jud A. Plener P. L. Brahler E. Brown R. C. (2018). Trends in child maltreatment in Germany: comparison of two representative population-based studies. Child Adolesc. Psychiatry Ment. Health 12:24. 10.1186/s13034-018-0232-5 29849753 PMC5970447

[ref211] Wolf R. C. Sambataro F. Vasic N. Schmid M. Thomann P. A. Bienentreu S. D.. (2011). Aberrant connectivity of resting-state networks in borderline personality disorder. J. Psychiatry Neurosci. 36, 402–411. 10.1503/jpn.100150, PMID: 21406160 PMC3201994

[ref212] Woller W. Leichsenring F. Leweke F. Kruse J. (2012). Psychodynamic psychotherapy for postraumatic stress disorder related to childhhod abuse – principles for a treatment manual. Bull. Menninger Clin. 76, 69–93. 10.1521/bumc.2012.76.1.69 22409207

[ref213] World Health Organization (2018). International statistical classification of diseases and related health problems (11th revision). Available at: https://icd.who.int/browse11/l-m/en (Accessed December 31, 2018).

[ref214] Zanarini M. C. Frankenburg F. R. Dubo E. D. Sickel A. E. Trikha A. Levin A.. (1998). Axis I comorbidity of borderline personality disorder. Am. J. Psychiatry 155, 1733–1739. 10.1176/ajp.155.12.1733, PMID: 9842784

[ref215] Zucker M. Spinazzola J. Blaustein M. Van der Kolk B. A. (2006). Dissociative symptomatology in posttraumatic stress disorder and disorders of extreme stress. J. Trauma Dissociation 7, 19–31. 10.1300/J229v07n01_03, PMID: 16618693

